# KRASG12D Cells Override Homeostatic Cell Elimination Mechanisms in Adult Pancreas Via Wnt5a and Cell Dormancy

**DOI:** 10.1053/j.gastro.2025.02.042

**Published:** 2025-04-07

**Authors:** Beatriz Salvador-Barbero, Markella Alatsatianos, Jennifer P. Morton, Owen J. Sansom, Catherine Hogan

**Affiliations:** 1 European Cancer Stem Cell Research Institute, School of Biosciences, Cardiff University, Cardiff, United Kingdom; 2 Cancer Research UK Scotland Institute, Glasgow, United Kingdom; 3 School of Cancer Sciences, University of Glasgow, Glasgow, United Kingdom

**Keywords:** Early Tumorigenesis, Pancreatic Cancer, Epithelial Homeostasis, Cell Extrusion, Cell Competition, Oncogenic *RAS*, Wnt5a, Cell Dormancy

## Abstract

**Background & Aims:**

The adult pancreas protects against cancer by actively expelling genetically mutated cells. Pancreatic cancer starts with cells carrying KRAS mutations; however, it is not clear how some KRAS mutant cells override cell elimination mechanisms to survive in tissues.

**Methods:**

An in vivo mouse model of sporadic tumorigenesis was used to induce *Kras* and/or *Tp53* mutations in low numbers of cells in the adult pancreas. The mutant cell fate was monitored over time using quantitative fluorescence imaging. Gene signatures of noneliminated mutant cell populations were identified using bulk RNA sequencing. Differential gene expression was overlapped with publicly available datasets. Key molecular pathways were validated in murine pancreas using immunofluorescence and functionally tested using inhibitor studies in vivo and epithelial coculture systems in vitro.

**Results:**

Although most genetically mutant cells are eliminated from the adult pancreas, a population of KRASG12D- or p53R172H-expressing cells are stably retained. Wnt5a signaling, cell dormancy, and stemness were identified as key features of surviving KrasG12D cells in vivo. Wnt5a specifically inhibits apical extrusion of RasV12 cells by promoting stable E-cadherin–based cell–cell adhesions at RasV12: normal cell–cell boundaries in vitro. In the pancreas, Wnt signaling, E-cadherin, and b-catenin are increased at cell–cell contacts between noneliminated KrasG12D cells and normal neighbors. Active Wnt signaling is a general mechanism required to promote KrasG12D and p53R172H cell retention and cell survival in vivo.

**Conclusions:**

RAS mutant cells activate Wnt5a and cell dormancy to avoid cell expulsion and to survive in the adult pancreas.

## Graphical abstract

**Figure:**
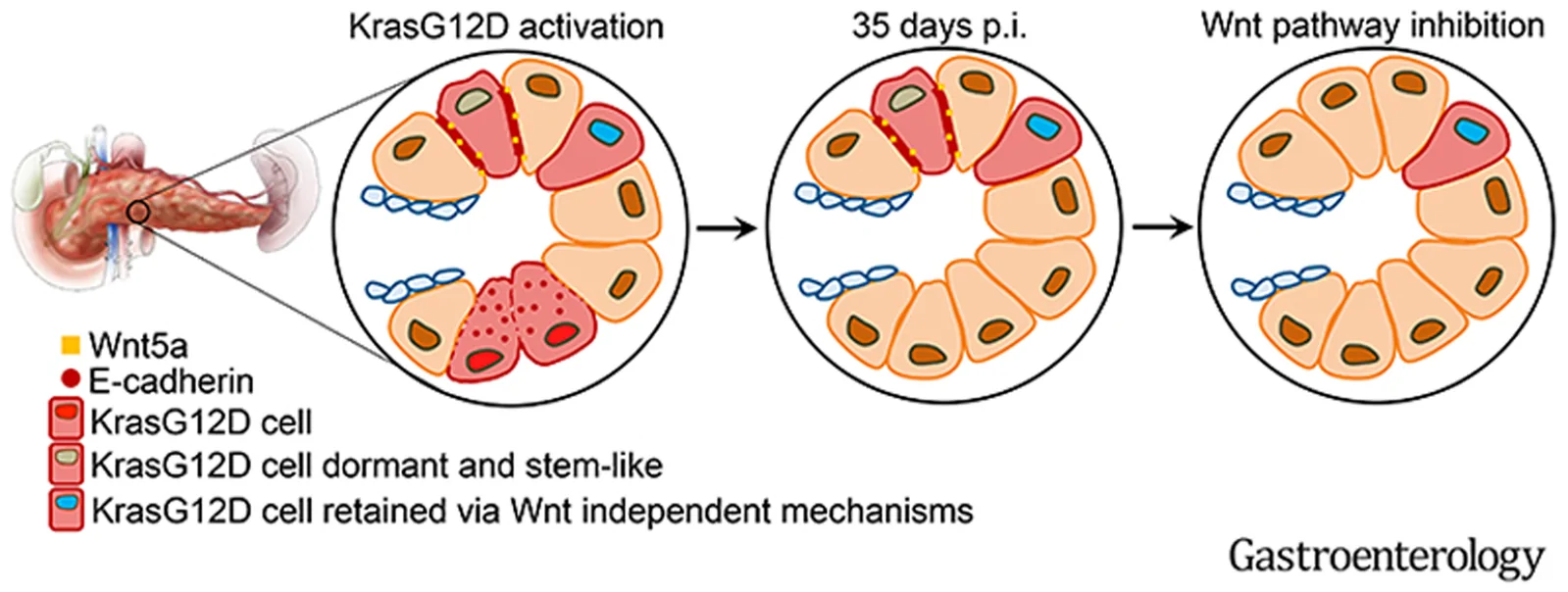

Epithelial tissues are continuously exposed to mutational insults and, as a result, genetically mutant cells often arise in a tissue. Most human cancers start sporadically from epithelial cells carrying genetic mutations that activate oncogenes or inactivate tumor suppressor function. Remarkably, epithelial tissues protect against tumorigenesis by actively removing genetically mutant cells.^1^ In general, genetically different cells compete for survival in tissues, resulting in the elimination of “less fit” mutant cells via apoptosis, extrusion, and/or cell differentiation.^1^ Under certain conditions,^2,3^ competition is tipped in favor of mutant cells. This is better understood in rapidly proliferating tissues that are actively replenished via defined stem cell compartments; tumor risk increases when stem cells acquire genetic mutations that confer a competitive advantage over wild-type counterparts.^4–6^ In contrast, our understanding of how competition outcomes shape tumorigenesis in slow proliferating adult tissues that lack a bona fide stem cell compartment remains poorly detailed.

In the pancreas, we recently demonstrated that cells expressing oncogenic Kras (Kras^G12D^) are outcompeted by healthy neighbors and are eliminated from exocrine and endocrine epithelial compartments.^7^ We have previously described how interactions with normal cells trigger robust evolutionary conserved cell biology phenotypes in *Ras* transformed cells, resulting in the elimination of the mutant cells via cell segregation and cell extrusion.^7–10^ Removal of KrasG12D cells from adult pancreas tissues requires local remodeling of E-cadherin–based cell–cell adhesions at normal–mutant boundaries and dynamic changes in mutant and normal cell volume.^7^ Importantly, we demonstrated that abrogation of KrasG12D cell elimination in the pancreas significantly increased the appearance of preneoplastic lesions,^7^ suggesting cell competition and the subsequent expulsion of mutant cells are disease preventative.

Activating mutations in oncogenic *RAS* is the principal driver gene event in human pancreatic ductal adenocarcinoma,^11^ the most common type of human pancreatic cancer. *KRAS* mutations are detected in >90% of human tumors and in vivo mouse studies have found that KRAS signaling is essential for disease to progress through all stages.^12^ Missense mutations in *TP53* are the second most common mutation detected in approximately 70% of human pancreatic ductal adenocarcinoma^13^ and required for metastasis.^14^ How genetically mutant cells survive and grow in the competitive environment of the adult pancreas remains unclear. We sought to understand the mechanisms underpinning how mutant cells avoid cell expulsion and survive in the adult pancreas.

## Methods

### Mouse Lines, Induction of Cre Recombinase In Vivo, Inhibition of Wnt Pathway In Vivo

Animals were housed in conventional pathogen-free animal facilities and experiments were conducted in accordance with UK Home Office regulations (ASPA 1986 and EU Directive 2010) under the guidelines of Cardiff University Animal Welfare and Ethics Committee. *Pdx1-Cre*^*ERT*^; *LSL-Kras*^*G12D/*+*15*^; *LSL-Trp53*^*R172H/*+16^; *Rosa26*^*LSL-tdRFP*17^ male and female 6- to 8-week-old mice were induced by intraperitoneal injection of tamoxifen in corn oil (low dose: 1 mg/40 g bodyweight once; megadose: three 9 mg/40 g injections^10^). Wnt signaling inhibition followed low-dose tamoxifen schedule; mice were aged to 35 days post induction (p.i.) and treated with WNT-974 (Stratech) 1.5 mg/kg or vehicle (dimethyl sulfoxide [DMSO]) in corn oil by oral gavage 5 d/wk for 4 weeks.

### RNA Sequencing

Sequenced reads Fastq files were quality checked using FastQC software (Babraham Bioinformatics). Reads were aligned to the mouse genome using the STAR package, and reads were counted using the FeatureCounts package, after removing duplicates using the MarkDuplicates tool (GATK). Differential expression was normalized and calculated using DESeq2 package, comparing different genotypes against control. Gene set enrichment analysis (GSEA) software, version 4.2.2 (Broad Institute) was used for pathway enrichment analysis and Prism software, version 10.0.2 (GraphPad) was used for heatmap graphs and normalized enrichment score graphs (GEO ID: GSE255283).

### Tissue Staining

Pancreas was harvested at specified time points and fixed in 10% neutral buffered formalin overnight at 4°C, before dissection into 2 pieces (head-body and body-tail) and embedded in either paraffin or OCT embedding matrix. Formalin-fixed paraffin-embedded (FFPE) pancreas was sectioned (7-mm thickness) and stained with anti-RFP, anti-cleaved caspase-3, and anti–Ki-67 with antibodies via immunohistochemistry protocols. Tissues sections were dewaxed and rehydrated. For antigen retrieval, tissue sections were incubated for 15 minutes at 37°C in 20 µg/mL Proteinase K diluted in Tris-buffered saline with Tween 20 or boiled in citrate buffer pH 6 for 15 minutes (Supplementary Table 14). Tissues were then blocked with 3% H_2_O_2_ for 20 or 10 minutes at room temperature (depending on the antigen retrieval method) and 5% normal goat serum (NGS) Tris-buffered saline with Tween 20 for 60 minutes at room temperature. Sections were incubated overnight at 4°C with the primary antibody diluted in 5% NGS Tris-buffered saline with Tween 20 (Supplementary Table 14). Tissues were stained with the secondary antibody ImmPRESS goat anti-rabbit (MP-7451, Vector Laboratories) for 60 minutes at room temperature, followed by 3,3^0^-diaminobenzidine tetra hydrochloride chromogen (peroxydase substrate kit, Vector Laboratories). Sections were dehydrated and mounted with DPX (Sigma-Aldrich). Immunofluorescence co-staining using either anti-CD44 and anti-RFP (Rockland) was performed in FFPE tissue slices using the same antigen retrieval and primary antibody incubations as described for immunohistochemistry, followed by Alexa Fluor secondary antibody incubation for 60 minutes at room temperature and Hoechst (ThermoFisher) (Supplementary Table 14) and mounted using Mowiol (Sigma-Aldrich).

For *β*-catenin, E-cadherin, K-cadherin, and RFP (Creative Diagnostics) immunofluorescence staining, pancreas was embedded in butyl-methyl methacrylate plastic under UV after dehydration and resin infiltration.^7,18^ Tissue was sectioned in 2-*µ*m-thick slices and rehydrated. Tissue staining followed the same protocol as immunofluorescence in FFPE sections.

Pancreas embedded in OCT embedding matrix was sectioned (10-*µ*m thickness), permeabilized with 0.05% sodium dodecyl sulfate (Sigma-Aldrich) and 0.05% Triton X100 (Sigma-Aldrich) solution, blocked with phosphate-buffered saline (PBS) with Tween 20 (0.01% Triton X100) and stained with anti-p27, anti-Wnt5a, anti-Dvl2 and anti-Sox9 primary antibodies for 2 hours at room temperature, Alexa Fluor secondary antibody incubation for 60 minutes at room temperature and Hoechst (Thermo-Fisher) and mounted using Mowiol (Sigma-Aldrich). Antibody details are detailed in Supplementary Table 14.

Global levels of endogenous RFP were measured in 10-*µ*m formalin-fixed OCT frozen sections, as described previously.^7^ RFP-positive area was averaged from 3 tissue slices per mouse separated by at least 20 *µ*m between each section.

To assess the presence of pancreatic intraepithelial neoplasia (PanIN) lesions, FFPE sections were stained with Alcian blue, as described previously.^7^ *β*-galactosidase activity was determined using the Senescence *β*-galactosidase Staining Kit (9860, Cell Signaling) using the manufacturer’s instructions.

### Madin-Darby Canine Kidney Cell Lines and In Vitro Experiments

For extrusion assays, Madin-Darby Canine Kidney (MDCK)-pTR GFP–RasV12 cells were combined with parental MDCK cells at a 1:50 ratio and induced using tetracycline, as described previously.^9,10^ For Wnt signaling experiments, GFP-RasV12 cells were treated with PBS, Wnt3a (1 *µ*g/mL), or Wnt5a (100 ng/mL) 2 hours post-tetracycline induction.

c-Myc was silenced in tetracycline-induced GFP-RasV12 cells by transfection with 100 ng si (small interfering) RNA oligos targeting *Myc* (see Supplementary Material). GFP-RasV12 cells were mixed with parental MDCK cells 24 hours after transfection and fixed after an additional 48 hours

For Wnt pathway inhibition, GFP-RasV12-expressing cells were transfected with siMyc1+2/siScr or treated with recombinant Wnt5a/PBS for 24 hours and then mixed with parental cells. Wnt5a treatment was maintained during the whole experiment. WNT-974 (Stratech, 1 *µ*M) or *OMP*-18R5 (10 mg/ mL) was added to the medium 8 hours after GFP-RasV12 and parental cells were mixed.

Confrontation assays and migration speed analysis were carried out as described previously.^10^ siMyc/siScr-GFP-RasV12 cells were plated 24 hours after transfection and inserts were removed 8 hours after. Cells were treated with PBS/Wnt5a for 8 hours before inserts were removed. PBS/Wnt5a treatment was maintained during the whole experiment. WNT-974/DMSO was added when inserts were removed.

### Pancreatic Ductal Epithelial Cell Co-Culture Assays

Harvesting and culture of nontransformed pancreatic ductal epithelial cells, cell–cell mixing, and immunostaining was carried out as described previously.^7^ Transformed tumor-derived epithelial cells obtained from KC mice (KrasG12D-expressing ductal epithelial cells) were treated with PBS or recombinant Wnt5a for 24 hours, before being prelabeled with CMFDA Green CellTracker dye (ThermoFisher Scientific) 1:1000 for 1 hour at 37°C. PBS/Wnt5a was maintained in the medium; 12 hours after plating cells were treated with DMSO or WNT-794 (Stratech, 1 *µ*M) for 36 hours. Cells were fixed and stain using anti–E-cadherin antibody (BD Biosciences).

### Statistical Tests

Statistical analyses were performed using Prism software, version 10.0.2. Normally distributed data, as determined by the Shapiro-Wilke test or D’Agostino and Pearson test were analyzed using unpaired Student *t* tests. A *P* value of < .05 was considered as significant and a rejection of the null hypothesis. Graphical data represent mean ± SD. Gene sets were considered enriched if they had a false discovery rate of <0.25. Heatmaps were created by computing normalized gene counts for each individual sample into row *z* scores.

Additional methods are described in Supplementary Material.

## Results

### Cells Expressing KrasG12D or p53R172H Mutations Are Eliminated From Adult Pancreas

**Figure 1:**
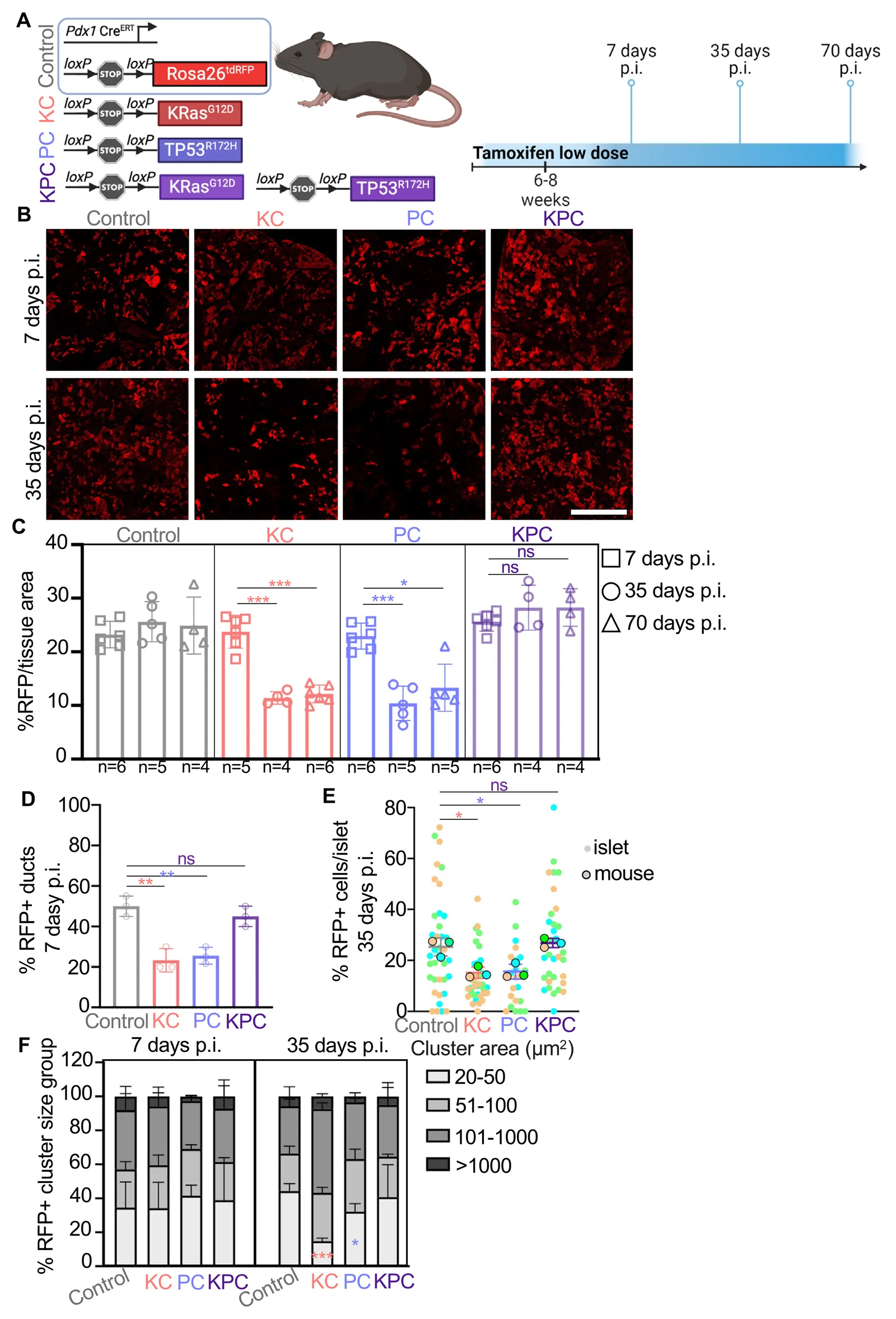
Cells expressing p53R172H are eliminated from adult pancreas in vivo. Coexpression of p53R172H mutations prevents KrasG12D cell elimination in vivo. (*A*) *Schematic* of mouse models (*left*), tamoxifen treatment and time points (*right*). (*B*) Representative *images* of endogenous RFP fluorescence. *Scale bar:* 500 *µ*m. (*C*) Percentage of RFP fluorescence per total tissue area. N = mouse numbers described in the graph. (*D*) Percentage of RFP^+^ ducts at 7 days p.i. (n = 3 mice). (*E*) Per-centage of RFP^+^ cells per total number of cells in islets at 35 days p.i. (n = 3 mice). (*F*) Percentage of RFP^+^ clusters grouped according to cluster area (*µ*m^2^). Control, KrasG12D (KC), p53R172H (PC), double mutant (KPC). Data represent mean ± SD. Student *t* tests were used to analyze the data. *P < .05; **P < .001; ***P < .0001.

**Co-expression of both mutations in the same cell abrogates cell elimination in vivo**. To model sporadic pancreatic cancer, we used the following pancreas-specific genetically engineered mouse models: KC: *Pdx1-Cre*^*ERT*^; *LSL-Kras*^*G12D/*+^; *Rosa26*^*LSL-tdRFP*^; PC: *Pdx1-Cre*^*ERT*^; *LSL-Trp53*^*R172H/*+^; *Rosa26*^*LSL-tdRFP*^; and KPC: *Pdx1-Cre*^*ERT*^; *LSL-Kras*^*G12D/*+^; *Trp53*^*R172H/*+^; *Rosa26*^*LSL-tdRFP*^. Our experimental control was *Pdx1-Cre*^*ERT*^; *Rosa26*^*LSL-tdRFP*^ mice (Figure 1A, *left*). Adult mice were treated with low-dose tamoxifen and aged for 7, 35, or 70 days p.i. (Figure 1A, *right*).^7^ To monitor cell fate, we measured RFP levels over time. Consistently,^7^ we found that low-dose tamoxifen induced stochastic RFP labeling in approximately 20%–25% of the tissue in all genotypes (Figure 1B and C). RFP fluorescence significantly decreased in KC and PC tissues, and it did not significantly change in double-mutant (KPC) tissues from 7 to 35/70 days p.i. (Figure 1B and C). Pancreas tissue histology was unaffected by low-level transgene expression (Supplementary Figure 1A). Because exocrine acinar cells comprise >90% of the adult pancreas, these data suggest that changes in global RFP fluorescence reflect changes in RFP^+^ acinar cell populations over time. RFP^+^ ducts were significantly less frequent in KC and PC tissues 7-day p.i. compared with control, while number of RFP^+^ ducts in KPC tissues was comparable to controls (Figure 1D). The percentage of RFP^+^ cells per islet significantly decreased in KC and PC tissues, whereas the frequency of RFP^+^ cells per islet in KPC tissues was comparable to wild-type controls (Figure 1E). We have previously shown that KrasG12D cells are competitively eliminated at cell boundaries with surrounding normal cells, resulting in the elimination of small clusters.^7,19^ The percentage of small clusters (<50 *µ*m^2^) significantly decreased in KC and PC tissues over time, while no significant differences were found in KPC tissues, compared with controls (Figure 1F), suggesting KPC cells do not respond to cell–cell interactions with normal cells. Thus, like KrasG12D-expressing cells,^7^ p53R172H single mutant cells are eliminated from all epithelial compartments. Cells expressing both KrasG12D and p53R172H mutations are not eliminated from endocrine/exocrine epithelial tissues.

### Transcriptional Profiles of Noneliminated Mutant Cells Indicate Activation of Pro-Survival Signals

We consistently found that approximately 10% of tissue area remains RFP-labeled in KC (KrasG12D) and in PC (p53R172H) tissues post the 35-day time point^7^ (Figure 1B and C), indicating that some mutant cells are not eliminated from the pancreas in vivo. We found rare Alcian blue–positive lesions^7^ in 6 of 9 KC mice at 168 days p.i. (Supplementary Figure 1B), suggesting some noneliminated KrasG12D cells progress to PanINs. No PanIN lesions were detected in PC tissues. PanIN lesions were detected more frequently in 5 of 5 KPC tissues (Supplementary Figure 1B) and 4 of 5 KPC mice developed tumors by 168 days p.i.

**Figure 2:**
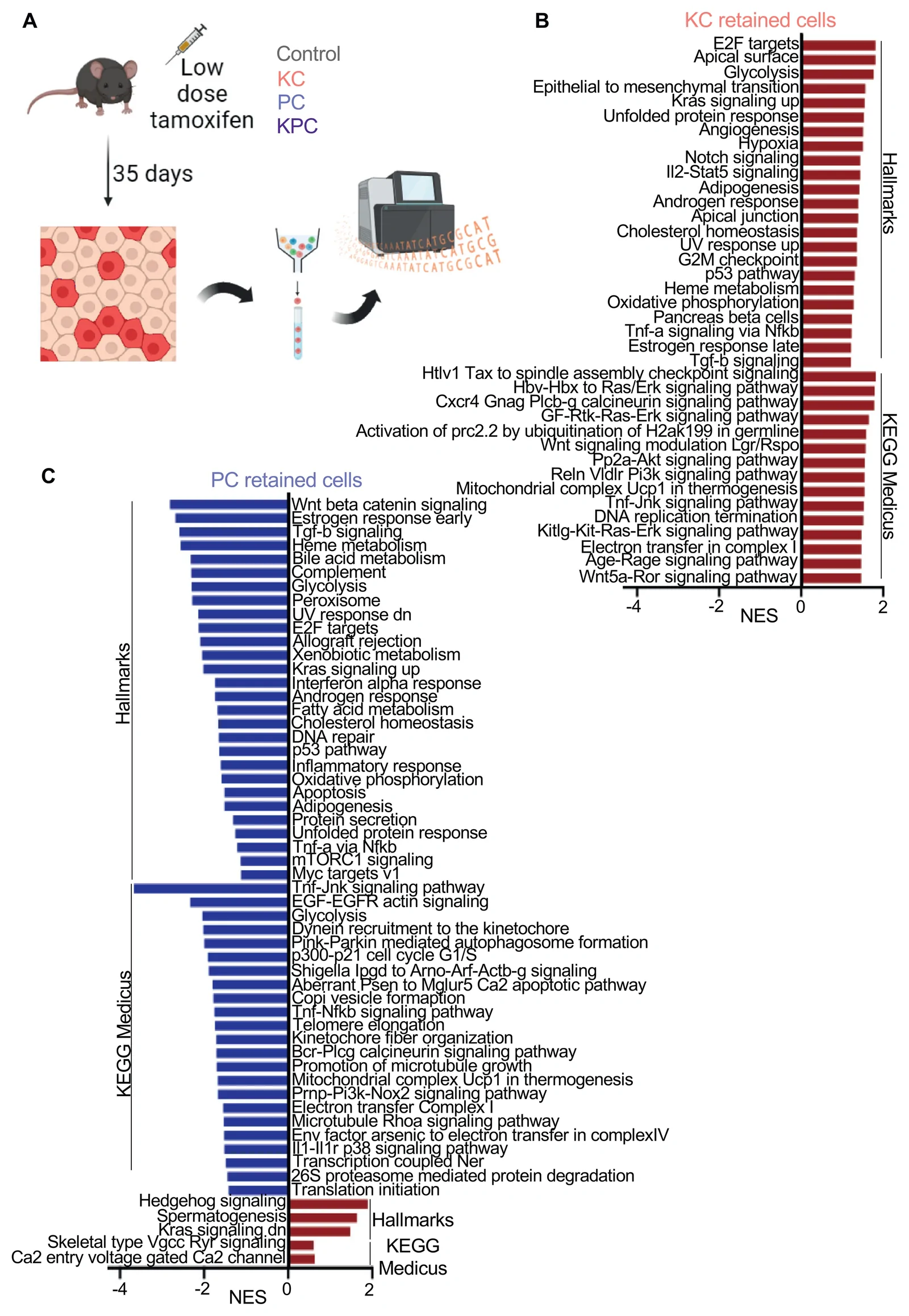
RNA sequencing results indicate cell fate changes in p53R172H and KrasG12D mutant retained cells. (*A*) Experimental design for tissue collection for RNA sequencing. (*B, C*) Normalized enrichment scores (NES; false discovery rate <0.25) of GSEA on the Hallmarks and KEGG MEDICUS gene sets for the RNA sequencing analysis of (*B*) KC retained cells or (*C*) PC retained cells. Full list of genes can be found in Supplementary Tables 4–7.

To identify gene signatures of noneliminated mutant cells and determine whether common pathways are activated by all mutant cells, we performed bulk RNA sequencing (Figure 2*A*). Whole pancreata were harvested from experimental cohorts (KC/PC/KPC/control) at 35 days p.i. We used anti-leptin antibodies to enrich for acinar/ ductal epithelial cells and fluorescence-activated cell–sorted leptin^+^ RFP^+^ cells for RNA sequencing (see Supplementary Methods). We assumed that most RFP^+^ cells isolated for RNA sequencing are acinar in origin as RFP^+^ ductal cells are rare at 7 days p.i. (Figure 1D). We compared differential gene expression in noneliminated mutant cells to wild-type controls. Unsupervised principal component analysis (Supplementary Figure 2A) and unsupervised clustering heatmaps of normalized differentially expressed genes (Supplementary Figure 2B) showed distinct transcriptional profiles for RFP^+^ cell populations of each genotype. In general, KC cells up-regulated genes, whereas PC and KPC cells down-regulated genes compared to wild-type controls (Supplementary Tables 1–3).

To elucidate the biological pathways underpinning mutant cell retention in pancreas tissues, we applied GSEA (GSEA Hallmarks, KEGG MEDICUS databases). We generated normalized enrichment scores with *P* values adjusted using false discovery rate of <0.25 to rank statistically significant gene sets enriched in KC, PC, or KPC transcriptomes compared to wild-type controls. KC, PC, and KPC (Figure 2B and C, Supplementary Figure 2C–F, 2G, Supplementary Tables 4–9) signatures deregulated Kras, MAPK, and p53 signaling pathways, suggesting deregulation of Kras signaling and/or p53 pathway is a general requirement for mutant cells to remain in tissues. KC signatures positively enriched for pro-survival and pro-tumorigenic pathways (eg, epithelial to mesenchymal transition, tumor necrosis factor–α signaling, hypoxia, angiogenesis, Notch signaling, and Wnt signaling) and immune response (eg, Il2-Stat5 signaling and tumor necrosis factor–*α* via nuclear factor *k*B signaling) (Figure 2B). Gene signatures from both PC (Figure 2C) and KPC (Supplementary Figure 2G) negatively enriched for apoptosis pathways and inflammatory responses, suggesting cell survival is a requisite for retention of mutant cells in tissues.

### Gene Signatures of Noneliminated KrasG12D Cells Correlate With Pancreatic Ductal Adenocarcinoma Initiation and With Increased Stemness and Cellular Reprogramming

**Figure 3:**
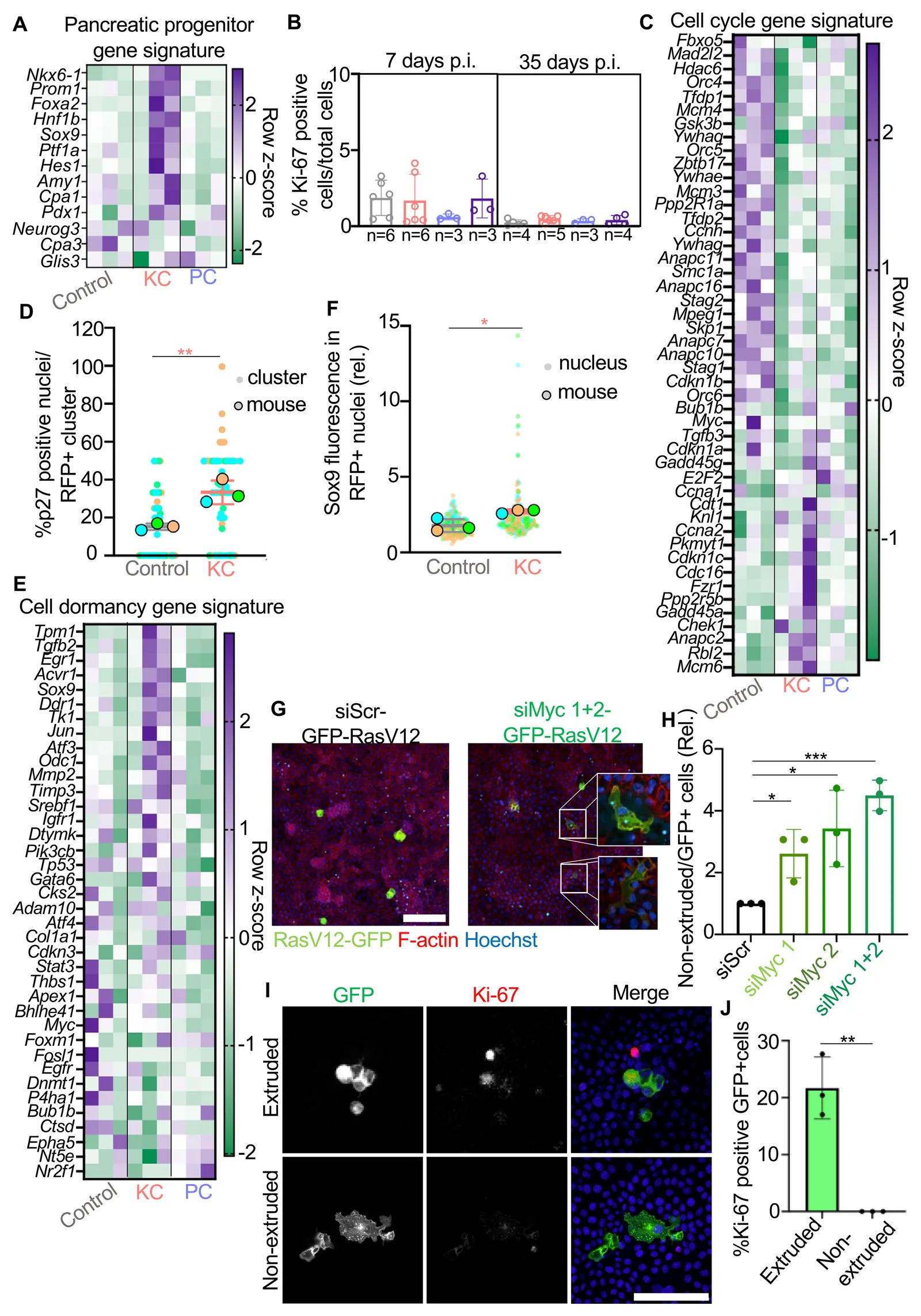
Cell dormancy/diapause gene signatures and cell cycle arrest are features of noneliminated Ras mutant cells. *Heatmaps* show row *z* scores of (*A*) pancreatic progenitor associated^23^ (*C*) cell cycle (KEGG M7963) associated and (*E*) cell dormancy^22^ gene signatures. Full list of genes can be found in Supplementary Table 10. (*B, D*) Percentage of Ki-67–positive cells/total cells. N = mouse numbers described in graph. (*D*) Percentage of p27 positive nuclei/total nuclei in each RFP^+^ cluster (n = 3 mice). (*F*) Mean fluorescence intensity relative to background of Sox9 per nuclei of RFP^+^ cells (n = 3 mice). (*G, I*) Confocal *images* of cell extrusion experiment. *Scale bar:* 100 µm. (*H*) Proportion of nonextruded GFP-RasV12 cells relative to nonextruded siScr-GFP-RasV12 cells (n = 3 experiments). (*J*) Percentage of Ki-67–positive cells extruded GFP-RasV12 cells of total extruded cells (n = 3 experiments). siScr, scrambled siRNA; siMyc1+2, two siRNA oligos targeting endogenous *Myc*. Data represent mean ± SD. Student *t* tests were used to analyze the data. *P < .05; **P < .001; ***P < .0001.

*KRAS* mutations are detected in the majority of human pancreatic ductal adenocarcinoma tumors and are required to drive all stages of pancreatic cancer.^12^ Thus, we focused our analyses on understanding how KrasG12D cells avoid cell elimination. Expression of oncogenic *Kras* in the pancreas triggers injury and stress responses, which often translate as cellular reprogramming and changes in cell fate.^20,21^ KC signatures positively correlated with WP_Pan-creatic_Adenocarcinoma_Pathway in GSEA analysis contrary to PC and KPC signatures (Supplementary Figure 2H–J). A consistent up-regulation of pancreas stem and progenitor genes (eg, *Nkx6-1, Prom-1, Hnf1b*)^22^ was observed in non-eliminated KC signatures (Figure 3A). Canonical markers of spasmolytic polypeptide-expressing metaplasia^23^ were up-regulated in noneliminated KC cells (Supplementary Figure 3A), as well as acinar, ductal, and mucin genes (Supplementary Figure 3B). Stemness gene signatures enriched in KC signatures compared with wild-type control, contrary to PC and KPC signatures (Supplementary Figure 3A–3I). Gene signature data imply that non-eliminated KrasG12D populations represent differentiated acinar cells and early embryonic pancreatic progenitors, cells undergoing acinar–ductal metaplasia and gastric pyloric and intestinal metaplasia, all of which are evident in tissues at very early time points.

### Noneliminated KrasG12D Mutant Cells Express Features of Cell Dormancy

Analysis of RFP levels in tissues over time (Figure 1C) indicated that noneliminated cells are not actively dividing in tissues. We found no evidence of cell senescence (*β*-galactosidase activity; Supplementary Figure 4A) or increased caspase-3–positive events (Supplementary Figure 4B) in 35-day KC pancreas tissues, suggesting a general absence of apoptosis. KC transcriptomes negatively correlated with oxidative stress response pathways (GOB-P_RESPONSE_TO_OXIDATIVE_STRESS, M3223; Supplementary Figure 4C). PC and KPC (Supplementary Figure 4D and E) gene signatures positively correlated with oxidative stress, suggesting noneliminated mutant p53-expressing populations (PC, KPC) are potentially under oxidative stress, whereas this is unlikely for KC cells. Immunostaining for Ki-67 showed an absence of proliferation in all tissues (Figure 3B). GSEA analyses identified enrichment of proliferation-related pathways (eg, E2F targets and GF-Rtk-Ras-Erk signaling) and enrichment of pathways indicative of dysregulation of the cell cycle towards genomic instability (eg, G_2_M checkpoint, Htlv Tax to spindle assembly checkpoint, Hbv-Hbx to Ras/Erk signaling, DNA-replication termination)^24^ in noneliminated KC cells compared to controls (Figure 2B). We observed a general down-regulation of cell cycle genes in KC signatures (Figure 3C, Supplementary Table 10). Similarly, gene expression profiles of PC cells (Figure 3C) and GSEA analyses of mutant p53-expressing cells indicated a general down-regulation of cell cycle and proliferation-related pathways (PC; Figure 2C, Supplementary Table 10; KPC; Supplementary Figure 2E). We scored a significant increase in p27-positive RFP^+^ nuclei in KC tissues compared to controls (Figure 3D), suggesting noneliminated KrasG12D cells are arrested at the G_1_ stage of the cell cycle.^25^ Interestingly, noneliminated KC gene signatures were also enriched for pathways associated with cancer cell dormancy (eg, oxidative phosphorylation, unfolded protein response, and hypoxia; Figure 2B). Cell dormancy describes a reversible cell cycle arrested state, often triggered by cell stress.^26^ Diapause is a temporary halt in embryogenesis when conditions are detrimental to development.^27^ Cell dormancy (Figure 3E, Supplementary Figure 4F) and diapause (Supplementary Figure 4G) gene signatures were enriched in noneliminated KC cells compared to control. Using Sox9 as a marker of dormant cells, we found that Sox9 fluorescence was significantly increased in RFP^+^ nuclei in KC tissues compared with RFP^+^ nuclei in wild-type controls (Figure 3F). NRF2 (a transcription factor activated during cell dormancy^28^) pathway and NRF2 target genes (Supplementary Figure 4H and I, Supplementary Table 11) positivelyenriched in non-eliminated KC cells only. Diapause gene signatures were not significantly enriched in PC cells (Figure 3E, Supplementary Figure 4G). These data infer that noneliminated mutant cells deregulate the cell cycle towards an arrested state; however, 2 copies of functional p53 are required for cells to adopt a dormant or diapaused state.^29–31^

### Cell Cycle Arrest Prevents Mutant Cell Extrusion In Vitro

We hypothesized that mutant cells override cell elimination in vivo by arresting in the cell cycle. To test this, we used previously established cell competition assays and MDCK cells expressing GFP-tagged constitutively active oncogenic RasV12 (GFP-RasV12).^8–10^ When surrounded by normal MDCK cells, GFP-RasV12–expressing MDCK cells are outcompeted via cell extrusion.^9,10^ In contrast, MDCK cells expressing mutant p53 are outcompeted by normal cells via necroptosis.^32^ c-Myc is a key regulator of the cell cycle and depletion of Myc triggers cell dormancy.^33^ We induced cell cycle arrest in GFP-RasV12 cells via *Myc* knockdown using 2 c-Myc siRNAs (siMyc-GFP-RasV12). Expression of c-Myc siRNA yielded a decrease in Myc and phospho-p38 protein levels, an increase in p21 protein levels (Supplementary Figure 5A and B) and a marked reduction in GFP-RasV12 cell confluency (Supplementary Figure 5C). In cell extrusion assays,^9,10^ GFP-RasV12 cells were first transfected with siScr or siMyc oligos and then mixed with normal MDCK cells at 1:50 ratios. The proportion of nonextruded GFP-RasV12 cells significantly increased when GFP-RasV12 cells were depleted for Myc (Figure 3G and H). Non-extruded siMyc-GFP-RasV12 cells were not proliferating, whereas the minority of Ki-67–positive cells were extruded (Figure 3I and J). We also used cell confrontation assays^10^ and live-cell imaging of normal–mutant interactions across an entire epithelial cell sheet (Supplementary Figure 5D). Upon collision with normal MDCK cells, GFP-RasV12 cells are triggered to retract and segregate away from normal cells, separating via the formation of smooth boundaries.^10^ GFP-RasV12 cells depleted for Myc (siMyc1+2) retracted less efficiently than GFP-RasV12 siScr controls (Supplementary Figure 5E and F). Thus, RasV12 cells depleted for Myc and exhibiting cell cycle arrest are not extruded or triggered to segregate by normal cells, suggesting cell cycle arrest protects RasV12 mutant cells from cell elimination.

### Noneliminated KrasG12D Cells Activate *β*-Catenin Independent Wnt Signaling In Vivo

**Figure 4:**
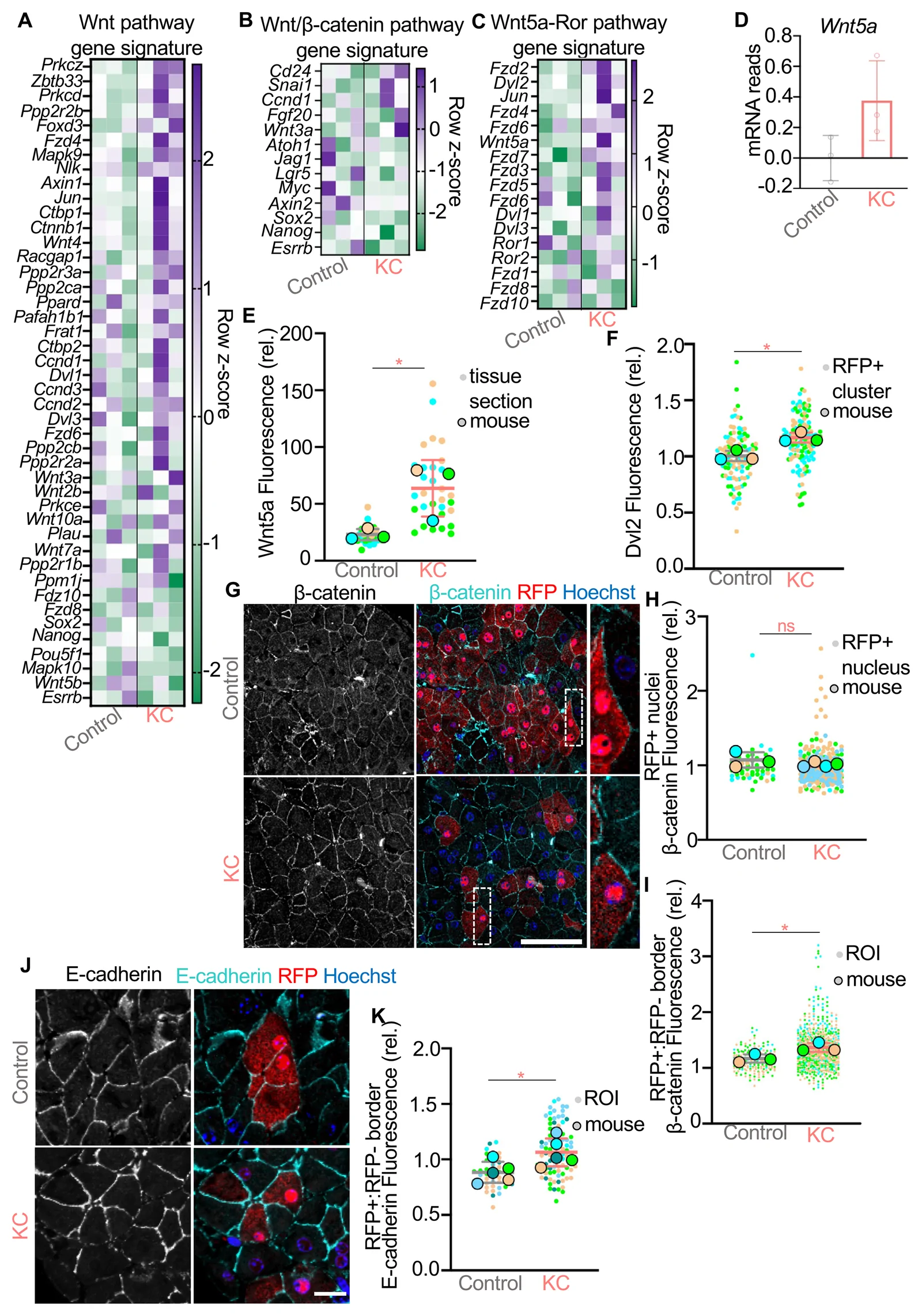
Wnt pathway is activated at normal–mutant cell–cell boundaries in vivo. *Heatmaps* show row *z* scores of (*A*) Wnt signaling pathway (WP_Wnt_signaling MM15829), (*B*) Wnt/*β*-catenin pathway (Hallmark_Wnt_beta_catenin_signaling MM3864) or (*C*) Wnt5a-Ror pathway (KEGG_Medicus_Wnt5a_Ror_signaling M47823) related genes. (*D*) *Wnt5a* messenger RNA reads relative to control reads obtained from the RNA sequencing experiment (n = 3 samples). (*E, F*) Mean fluorescence intensity relative to background of Wnt5a (*E*) or Dvl2 (*F*) (n = 3 mice). (*G*) Representative images of RFP and *β*-catenin staining. *Scale bar*: 50 *µ*m. (*H, I*) Mean fluorescence intensity relative to background of *β*-catenin in the nuclei of RFP^+^ cells (*H*) or at the boundary between RFP^+^:RFP^–^ cells (*I*) (n = 3 mice). (*J*) Confocal *images* of pancreas tissues stained with anti-RFP and anti–E-cadherin. *Scale bar:* 20 *µ*m. (*K*) Mean fluorescence intensity relative to background of E-cadherin at the boundary between RFP^+^:RFP^–^ cells (n = 5 mice). Data represent mean ± SD. Student *t* tests were used to analyze the data. *P < .05.

The Wnt pathway positively regulates cancer cell dormancy,^34,35^ diapause,^36^ and stemness.^37^ GSEA analyses identified an enrichment for Wnt pathway activation in noneliminated KC transcriptomes compared to controls (Wnt signaling modulation Lgr/Rspo, Wnt5a-Ror signaling pathway) (Figure 2B) and increased expression of Wnt pathway–related genes (Figure 4A). Wnt/*β*-catenin pathway gene expression and signatures were generally down-regulated in KC and PC cells (Figures 2C and 4B, Supplementary Figure 6A, Supplementary Table 12). Both KC and PC gene signatures positively correlated with Wnt5a-Ror signaling pathway (Supplementary Figure 6B and C), and Wnt ligands and receptors associated with Wnt5a-Ror signaling were increased in KC cells (Figure 4C and D, Supplementary Table 12).

To investigate active Wnt signaling in vivo, we first analyzed expression of CD44 protein, a target of *β*-catenin– dependent and independent signaling.^38^ Consistently,^39^ we observed CD44 labeling at cell membranes in PanINs (Supplementary Figure 6D). CD44 was significantly increased at RFP^+^:RFP^–^ cell–cell boundaries in KC and PC tissues compared to wild-type controls (Supplementary Figure 6E. F, and H). In contrast, CD44 was significantlyN reduced within a cluster of KC and PC cells (Supplementary Figure 6E, G, and I). We compared CD44 levels in tissues where KrasG12D cells are not competitively eliminated (high dose of tamoxifen-KC megadose)^7^ and found CD44 levels were significantly lower at mutant–normal cell boundaries in KC megadose tissues compared to KC low-dose tissues (Supplementary Figure 6E and F), and signifi-cantly higher between KC cells (Supplementary Figure 6E and G). We observed significant increase in Wnt5a (Figure 4E) and Dvl2 (Figure 4F) protein in KC cells relative to controls, suggesting Wnt5a signaling is active in non-eliminated KrasG12D cells in vivo. Nuclear *β*-catenin was undetectable in both control and KC cells (Figure 4G and H), indicating that Wnt/*β*-catenin signaling is not active in noneliminated KC cells, consistent with our transcriptional analysis. Instead, *β*-catenin was significantly increased at RFP^+^:RFP^–^ cell–cell contacts in KC tissues compared to controls (Figure 4G and I). Moreover, E-cadherin was also significantly elevated at RFP^+^:RFP^–^ boundaries in KC tissues compared to controls (Figure 4J and K). We also detected increased messenger RNA expression of atypical *cadherin-6* (Supplementary Figure 7A) and increased protein levels (K-cadherin) in KC cells (Supplementary Figure 7B and C). Together, our data suggest that *β*-catenin–independent Wnt signaling is active in vivo and Wnt signaling increases cohesiveness and/or stability of E-cadherin-based cell–cell adhesions at cell–cell boundaries between noneliminated KrasG12D cells and normal neighbors.

### Wnt5a Inhibits RasV12 Cell Extrusion In Vitro by Stabilizing E-Cadherin–Based Cell–Cell Adhesions

**Figure 5:**
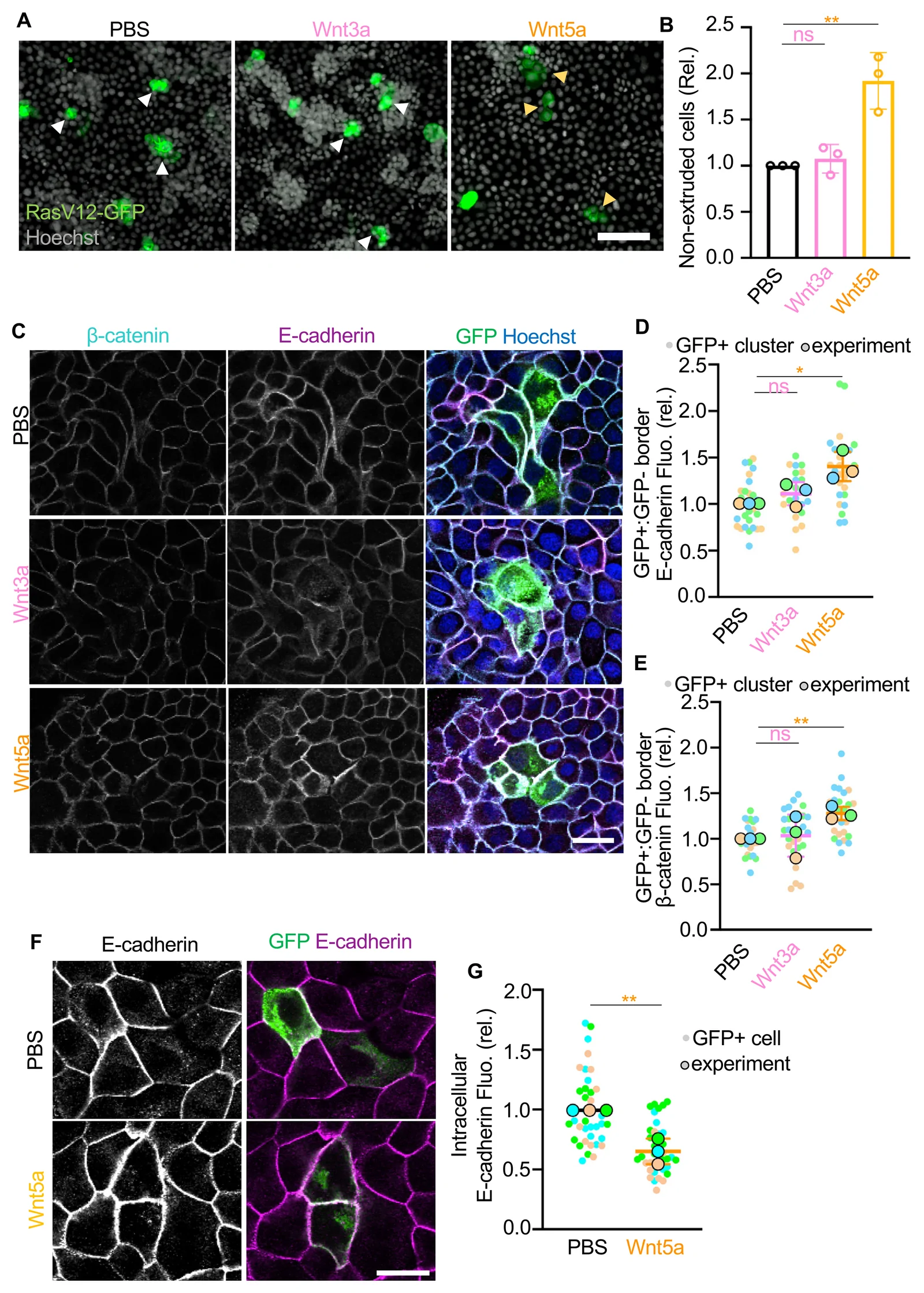
Wnt5a induces stable E-cadherin–based cell–cell adhesion in RasV12 cells. (*A*) Confocal images of cell extrusion experiment. *Scale bar:* 100 *µ*m. (*B*) Proportion of nonextruded GFP-RasV12 cells relative to nonextruded PBS-treated GFP-RasV12 cells (n = 3 experiments). (*C, F*) Confocal *images* of GFP-RasV12 cells mixed with MDCK cells 48 hours (*C*) or 16 hours (*F*) after treatment. *Scale bar:* 20 *µ*m. (*D, E*) Mean fluorescence intensity of E-cadherin (*D*) or *β*-catenin (*E*) at the boundary between GFP-RasV12 and MDCK cells relative to PBS-treated cells (n = 3 experiments). (*G*) Mean fluorescence intensity of intracellular E-cadherin in GFP-RasV12 relative to PBS-treated cells (mixed with MDCK cells; n = 3 experiments). Data represent mean ± SD. Student *t* tests were used to analyze the data. *P < .05; **P < .001.

To establish whether Wnt5a directly inhibits RAS mutant cell elimination, we returned to MDCK cell extrusion assays.^9,10^ The proportion of nonextruded GFP-RasV12 cells significantly increased in the presence of Wnt5a compared with PBS-treated controls (Figure 5A and B). Remarkably, addition of Wnt3a had no effect on RasV12 cell extrusion rates (Figure 5A and B), suggesting only *β*-catenin– independent Wnt signaling inhibits RasV12 cell extrusion.

Cell extrusion requires dynamic remodeling of E-cadherin–based cell–cell adhesions at normal–mutant cell–cell boundaries.^9,40,41^ E-cadherin and *β*-catenin staining was more defined and significantly increased at normal–mutant cell–cell contacts in Wnt5a-treated cells compared to PBS-treated controls (Figure 5C–E). In contrast, Wnt3a treatment had no significant effect on E-cadherin or *β*-catenin at RasV12–normal cell–cell contacts; instead, E-cadherin/*β*-catenin was diffuse/weak and sometimes absent, like controls (Figure 5D and E). Treatment with Wnt5a significantly increased the levels of E-cadherin (Supplementary Figure 7D), but had no effect on *β*-catenin levels (Supplementary Figure 7E) detected at cell–cell contacts between RasV12 cells, suggesting Wnt5a signaling specifically modulates E-cadherin at cell–cell contacts. Addition of Wnt3a had no statistically significant effect on E-cadherin or *β*-catenin at cell–cell contacts between RasV12 cells (Supplementary Figure 7D and E). In cell confrontations assays,^10^ Wnt5a treatment significantly reduced RasV12 cell speed compared to PBS-treated controls, whereas normal MDCK cell speed was unaffected (Supplementary Figure 7F), implying Wnt5a signaling specifically affects RasV12 cell cohesion.

E-cadherin endocytosis is required for apical extrusion of RasV12 cells from normal MDCK cell sheets.^41^ Before cell extrusion events (16 hours),^41^ E-cadherin staining was weakly visible/diffuse at RasV12–normal cell–cell contacts in PBS-treated controls and was often detected in distinct intracellular puncta in both RasV12 and normal cells (Figure 5*F*). In contrast, Wnt5a-treated cells showed strong, defined E-cadherin at cell–cell contacts and E-cadherin– positive puncta were less visible in RasV12 cells and neighboring cells, which was reflected in a significant reduction in fluorescence intensity (Figure 5F and G). Caveolin-1 (a key regulator of endocytosis and cell extrusion^42^) fluorescence was significantly reduced at RasV12– normal cell–cell contacts in Wnt5a-treated cells compared with PBS controls (Supplementary Figure 7G and H), suggesting Wnt5a treatment blocks E-cadherin recycling/ endocytosis at RasV12–normal boundaries. Together, our data showed that Wnt5a stabilizes E-cadherin–based cell–cell adhesion at normal–mutant boundaries potentially by preventing E-cadherin internalization, which correlates with a significant reduction in RasV12 cell extrusion and increase in RasV12 cell cohesion.

### Wnt Signaling Is Required to Promote Retention of Mutant Cells In Vitro and In Vivo

**Figure 6:**
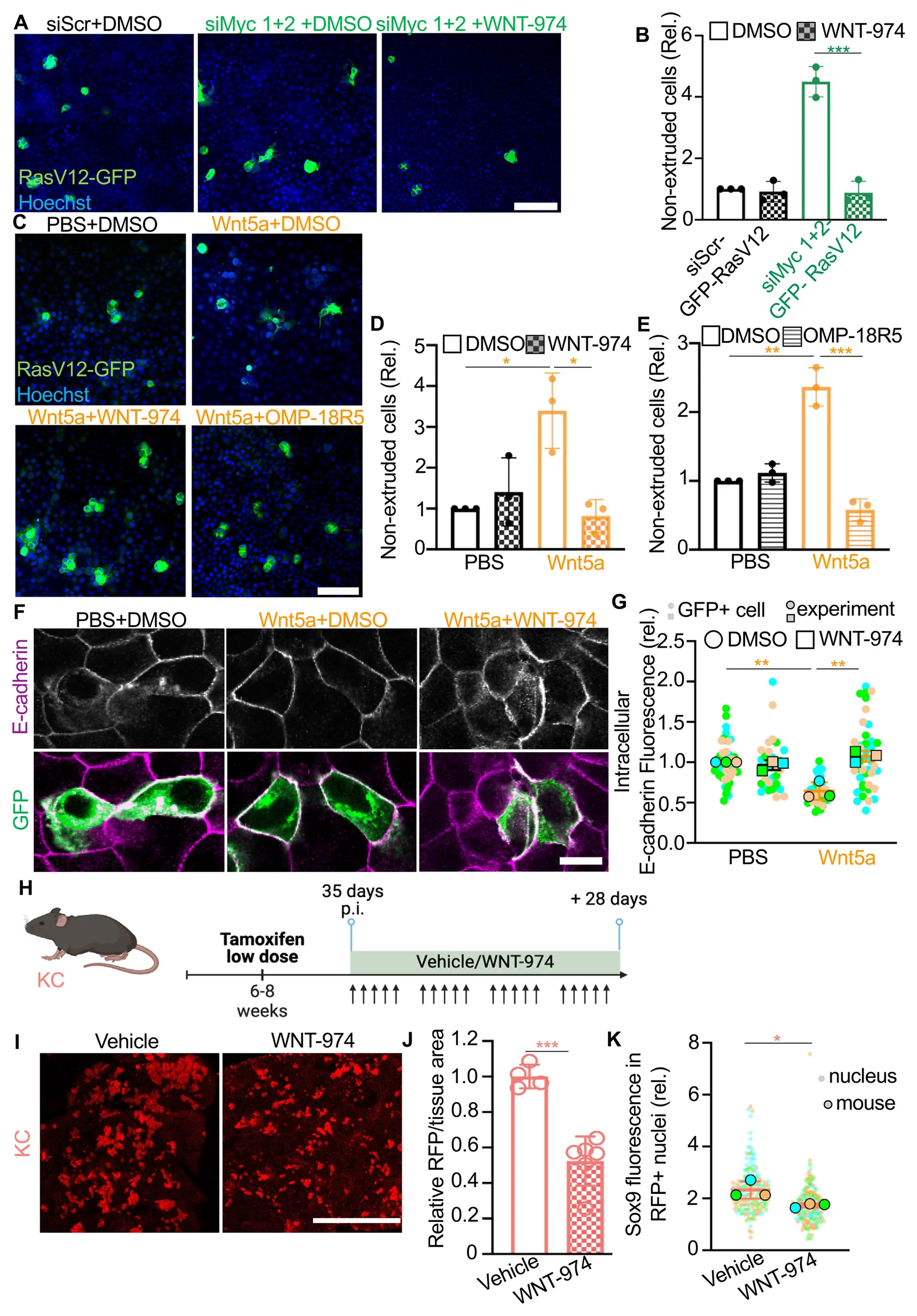
Wnt signaling prevents apical extrusion of RasV12 cells from normal epithelial monolayer in vitro. Inhibition of Wnt promotes elimination of noneliminated cells in vivo. (*A, C*) Confocal images of cell extrusion experiment, GFP-RasV12 transfected with siMyc/siScr (*A*) or treated with PBS/Wnt5a (*C*) and DMSO/WNT-974 for 48 hours. *Scale bar:* 100 *µ*m. (*B*) Proportion of nonextruded GFP-RasV12 transfected in (*A*) relative to siScr-GFP-RasV12 (n = 3 experiments). (*D, E*) Proportion of GFP-RasV12 nonextruded cells treated with DMSO/WNT-974 (*D*) or DMSO/OMP-18R5 (*E*) relative to PBS-/DMSO-treated cells. (*F*) Confocal *images* of GFP-RasV12 cells mixed with MDCK cells treated with PBS/Wnt5a and DMSO/WNT-974 for 16 hours. *Scale bar:* 20 *µ*m. (*G*) Mean fluorescence intensity of intracellular E-cadherin in GFP-RasV12 in F relative to PBS-/ DMSO-treated cells (n = 3 experiments). (*H*) Experimental design for Wnt inhibition experiments in vivo. (*I*) Representative *images* of endogenous RFP fluorescence in KC pancreas harvested 28 days post-vehicle/WNT-974-treatment. *Scale bar:* 500 *µ*m. (*J*) RFP^+^ area per total tissue area relative to vehicle (vehicle n = 4 mice; WNT-974 n = 5 mice). (*K*) Mean fluorescence intensity relative to background of Sox9 per nuclei of RFP^+^ cells in tissues treated with vehicle/WNT-974 (n = 3 mice). Data represent mean ± SD. Student *t* tests were used to analyze the data. *P < .05; **P < .001; ***P < .0001.

Next, we set out to test whether Wnt signaling is required to prevent mutant cell elimination. In MDCK cells, overexpression of RasV12 induces expression and secretion of Wnt5a in a porcupine-dependent manner.^43^ Porcupine is an acyltransferase enzyme required for the lipidation and trafficking of Wnt proteins.^44^ We observed a significant reduction in the proportion of nonextruded siMyc 1+2-GFP-RasV12 cells from normal monolayers treated with porcupine inhibitor WNT-974, compared to DMSO-treated co-cultures (Figure 6A and B). In cell confrontation assays,^10^ WNT-974–treated siMyc 1+2-GFP-RasV12 cells efficiently retracted and segregated from normal cells with a smooth boundary (Supplementary Figure 8A–C), compared with DMSO-treated cells.

The effects of Wnt5a on RasV12 cell extrusion rates were reversed in the presence of WNT-974 (Figure 6C and D) or the Frizzled receptor antagonist OMP-18R5^39^ (Figure 6C and E) compared with DMSO-treated controls. Wnt5a induced a significant decrease in the level of intra-cellular E-cadherin fluorescence in RasV12 cells, which was rescued in the presence of WNT-974 (Figure 6F and G), implying that Wnt signaling is required to prevent E-cadherin internalization and endocytosis. In cell confrontation assays, Wnt5a treatment significantly decreased RasV12 cell speed, and this significantly increased in the presence of WNT-974 (Supplementary Figure 8D). We repeated coculture experiments using primary murine pancreatic ductal epithelial cells.^7^ When mixed with nontransformed pancreatic ductal epithelial cells, prelabeled KRasG12D-expressing ductal epithelial cells are often visible on the apical surface of the underlying nontransformed cells (Supplementary Figure 8E). Treatment with Wnt5a signifi-cantly increased the number of KrasG12D-expressing cells integrated into the monolayer (Supplementary Figure 8E and F). This effect of Wnt5a was significantly reduced in the presence of WNT-974 (Supplementary Figure 8E and F). Consistent with MDCK experiments, Wnt5a treatment induced a significant reduction in the level of intracellular E-cadherin fluorescence in KrasG12D ductal epithelial cells, which was reversed after treatment with WNT-974 (Supplementary Figure 8G and H).

To functionally test whether active Wnt signaling is necessary for mutant cell retention in vivo, we first induced low-level recombination in adult KC or PC tissues, waited for 35 days p.i. for most mutant cells to be eliminated, and then administered WNT-974 or vehicle for 4 weeks (Figure 6H). CD44 fluorescence significantly decreased in WNT-974–treated KC tissues compared with vehicle (Supplementary Figure 9A), suggesting Wnt activity is reduced after treatment. We measured a significant decrease in RFP fluorescence in the WNT-974–treated KC tissues (Figure 6I,J) and in PC tissues (Supplementary Figure 9B and C) compared to vehicle-treated controls. Thus, inhibition of Wnt pathway induces the expulsion of KrasG12D or p53R172H cells from pancreas tissues in vivo, suggesting Wnt signaling is required to promote mutant cell retention. Notably, we observed a significant decrease in Sox9 fluorescence in RFP^+^ nuclei (Figure 6K) and a significant decrease in percentage of p27-positive cells (Supplementary Figure 9D) in KC tissues treated with WNT-974, compared to KC tissues treated with vehicle. These data suggest that active Wnt signaling is required to pro-mote cell dormancy/cell stemness in some KrasG12D cells in vivo. Our data support a model (Supplementary Figure 9E) whereby activation of Wnt signaling allows mutant cells to switch to a dormant cell state, avoid cell expulsion, and survive in the pancreas.

### High WNT5A Expression Correlates With Pancreatic Cancer Progression and Poor Prognosis in Humans

**Figure 7:**
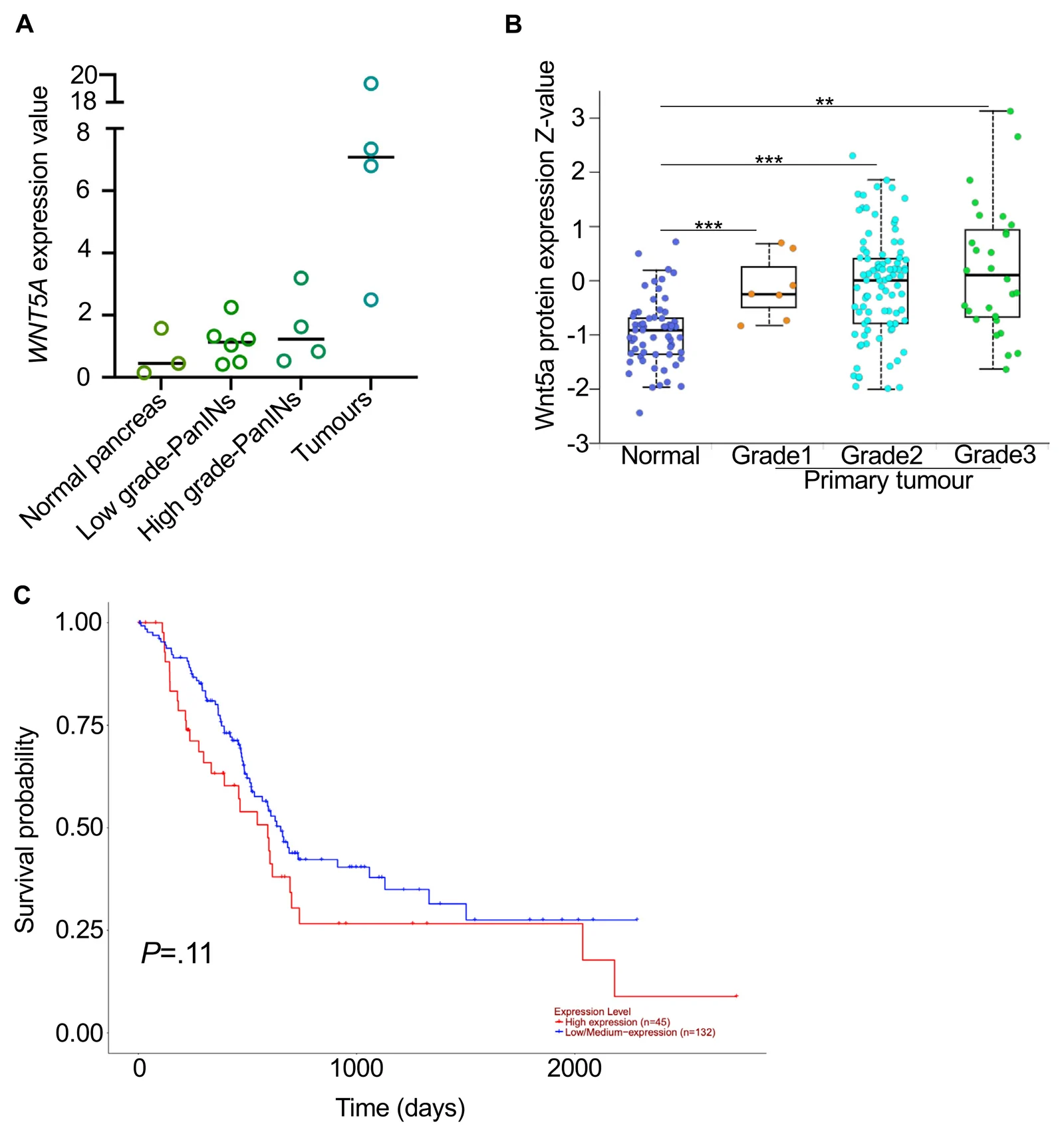
WNT5A protein levels are increased in human pancreatic adenocarcinoma compared to normal human pancreas. (*A*) *WNT5A* expression levels in normal pancreas, low-grade PanINs, high-grade PanINs and tumors (GSE210351). (*B*) The *z* value of WNT5A protein in normal pancreas and pancreatic adenocarcinoma. (*C*) Kaplan-Meier *graph* showing probability of survival depending on high or low *WNT5A* expression levels. **P < .001; ***P < .0001.

To translate our findings to the clinical setting, we mined publicly available RNA sequencing^45^ and proteomic datasets (Clinical Proteomic Tumour Analysis Consortium, The Cancer Genome Atlas Program). *WNT5A* (Figure 7A), *DVL2* (Supplementary Figure 10A), *FRIZZLED7* (FZD7, Supplementary Figure 10B) gene expression levels were elevated in PanIN lesions and increased pancreatic tumors compared to normal tissues. Expression of WNT5A protein was significantly increased in tumors of increasing grade compared to normal tissues (Figure 7B, Supplementary Figure 10C). High expression of *WNT5A* correlated with poor overall survival compared to medium/low expression (Figure 7C, *P* = .1). FZD7 (Supplementary Figure 10D) significantly increased at the protein level in human pancreatic adenocarcinoma samples compared to normal tissues. We conclude that elevated expression of *WNT5A* and high WNT5A signaling are strongly associated with human pancreatic cancer.

## Discussion

Tissue homeostasis is fundamental to healthy aging of an organism and cell competition is an important regulator of tissue health. Here, we extend our previously published results^7^ to show that, like KrasG12D-expressing cells, p53R172H-expressing cells compete with normal cells for survival in the adult pancreas and are often expelled from the tissue. We report that cell expulsion is inefficient, and a proportion of KrasG12D- or p53R172H-expressing cells are not eliminated from the tissue, suggesting these cells have a survival advantage. We found that Wnt5a signaling is active in noneliminated cells and directly inhibits cell elimination mechanisms both in vitro and in vivo, promoting mutant cell survival. We found that coexpression of p53R172H and KrasG12D in the same cell (KPC) provides all mutant cells with a survival advantage and “double-mutant” KPC cells are not eliminated, consistent with previous studies.^32,46^

Apical cell extrusion is an active process that requires direct interaction with normal cells.^9^ E-cadherin is a key modulator of apical cell extrusion,^7,9,40,41^ suggesting E-cadherin must be dynamically remodeled for cell extrusion to occur. We previously showed that E-cadherin is decreased at KrasG12D–normal cell–cell adhesions in vivo, specifically at time points before KrasG12D cell elimination,^7^ and is predominantly intracellular in KrasG12D-expressing ductal epithelial cells surrounded by normal ductal epithelial cells in vitro.^7^ Here, we extended these findings to show that E-cadherin and *β*-catenin are increased specifically at cell–cell contacts between KrasG12D cells that are not eliminated and normal neighbors in vivo. Using MDCK and primary pancreatic ductal epithelial cell systems, we showed that treatment with Wnt5a induced a significant decrease in the level of intracellular E-cadherin detected in RAS cells. Instead, E-cadherin and *β*-catenin are increased at RAS–normal cell–cell contacts. Moreover, inhibition of Wnt signaling restores appearance of intracellular E-cadherin in RAS cells and apical extrusion events. Together our data support a model whereby stable cell–cell adhesion induced by Wnt5a signaling prevents cell extrusion. We also found that caveolin-1, an important driver of RasV12 cell extrusion,^42,47^ is also decreased at RasV12–normal cell–cell contacts after Wnt5a treatment. In development, Wnt5a signaling regulates cell cohesion and tissue fluidity via caveolin-dependent and clathrin-dependent endocytosis.^48^ In human epithelial cells, Wnt5a induces *β*-catenin membrane localization and association with E-cadherin, increasing intercellular adhesion.^49^ Whether Wnt5a directly modulates E-cadherin endocytosis/recycling and/or stability of E-cadherin at the membrane at RasV12–normal cell–cell boundaries requires further investigation.

RasV12 cells in a cell cycle arrested state were not extruded from MDCK monolayers. In pancreas, non-eliminated KrasG12D cells stained positive for p27, and up-regulated cell dormancy signatures and pathways known to induce cell dormancy.^50^ Thus, exiting the cell cycle protects KrasG12D cells from cell elimination in vivo and in vitro. Increased glycolysis and oxidative phosphorylation are also associated with pancreatic cancer stem cells.^51,52^ Indeed, our data indicate that some noneliminated KrasG12D cells adopt progenitor and/or stem cell characteristics, suggesting dormant cells are also stem-like cells and may represent a cancer cell of origin. Future studies are required to determine whether normal–mutant interactions in the adult pancreas induce cellular stress responses in genetically mutant cells and whether this in turn activates cell dormancy. Interestingly, Wnt5a has been shown to induce and maintain cancer cell dormancy in metastatic niches by negatively regulating canonical Wnt/*β*-catenin signaling.^34,35^ An exciting future direction of this work will be to elucidate whether the cell dormancy phenotype described here is regulated via Wnt-dependent mechanisms described for dormant metastatic cancer cells. Here, we show that inhibition of Wnt restores cell extrusion and cell segregation of cell cycle arrested RasV12 cells from normal cells in vitro. Sox9 levels in RFP^+^ nuclei significantly decreased and percentage of p27-positive KrasG12D cells are significantly reduced in WNT-974–treated tissues, suggesting a mechanistic link between active Wnt and cell dormancy/cell stemness in vivo is plausible.

*KRAS* is a key driver of pancreatic ductal adenocarcinoma. Single-cell profiling of pancreatic tumorigenesis in mouse models demonstrates that *Kras* mutant cells are very heterogeneous and show signatures of tumorigenesis even before premalignant lesions are established.^20^ Our RNA sequencing data confirmed this early transformation and cellular heterogeneity profile of KrasG12D cells. Pancreatic cancer is a devastating disease, which is generally diagnosed at late and incurable stages. An improved understanding of the biology of how pancreatic cancer starts and grows in adult tissues will inform the development of new early-detection tools. Our results challenge current understanding of how cancers start in the adult pancreas and suggest that genetically mutant cells must override tissue homoeo-stasis mechanisms to survive in tissues, before malignant transformation/expansion.

## Supplementary Material

Note: To access the supplementary material accompanying this article, visit the online version of *Gastroenterology* at www.gastrojournal.org, and at https://doi.org/10.1053/j.gastro.2025.02.042.

Supplementary Table 1

Supplementary Table 2

Supplementary Table 3

Supplementary Table 4

Supplementary Table 5

Supplementary Table 6

Supplementary Table 7

Supplementary Table 8

Supplementary Table 9

Supplementary Table 10

Supplementary Table 11

Supplementary Table 12

Supplementary Table 13

Supplementary Methods

## Data Availability

The RNA-sequencing data generated during this study are available at GEO ID: GSE255283 (https://eur03.safelinks.protection.outlook.com/?url=https%3A%2F%2Fwww.ncbi.nlm.nih.gov%2Fgeo%2Fquery%2Facc.cgi%3Facc%3DGSE255283&data05%7C02%7Csalvadorb%40cardiff.ac.uk%7Ce8683ba7b2c64f0a3bce=08dc280f1f5c%7Cbdb74b3095684856bdbf06759778fcbc%7C1%7C0%7C638429292617229243%7CUnknown%7CTWFpbGZsb3d8eyJWIjoiMC4wLjAwMDAiLCJQIjoiV2luMzIiLCJBTiI6Ik1haWwiLCJXV.CI6Mn0%3D%7C0%7C%7C%7C&sdata=5jxRazg7lAf2W2wqa%2Bwfa7kxPY9HVX01VXHr4PZQVA0%3D&reserved=0).

## What You Need To Know

### Background And Context

The adult pancreas actively eliminates KRAS-expressing cells, preventing the occurrence of preneoplasia. KRAS mutations are required at all stages of pancreatic cancer; however, it is not clear how some KRAS mutant cells override cell elimination mechanisms to survive

### New Findings

Activation of Wnt5a pathway and cell dormancy prevent KRAS cell expulsion from the tissue. Wnt5a signaling promotes stable cell–cell adhesions between KRAS mutant cells and normal neighbors, allowing mutant cells to be stably retained in the tissue.

### Limitations

Future studies are needed to determine the upstream regulators of Wnt5a and cell dormancy.

### Clinical Research Relevance

Inhibition of Wnt5a and/or disruption of cell dormancy could provide new strategies to prevent pancreatic tumour initiation and promote tissue health. By understanding the biology of these initial stages of disease will lead to the development of new early detection tools/biomarkers for pancreatic cancer.

### Basic Research Relevance

Genetically mutant cells activate Wnt5a and cell dormancy to override homeostatic tissue controls, which would otherwise rid tissues of mutant cells. Activation of Wnt5a and a dormant cell state occurs early in cancer development, before preneoplastic growth, to promote survival of cancer-causing cells in a competitive tissue environment.

## Abbreviations used in this paper

DMSO: dimethyl sulfoxide
FFPE: formalin-fixed, paraffin-embedded
GSEA: gene set enrichment analysis
MDCK: Madin-Darby Canine Kidney
NGS: normal goat serum
PanIN: pancreatic intraepithelial neoplasia
PBS: phosphate-buffered saline
p.i.: post induction
si: small interfering
