# Supplementary material for: KRASG12D Cells Override Homeostatic Cell Elimination Mechanisms in Adult Pancreas Via Wnt5a and Cell Dormancy
Source: Gastroenterology. Author manuscript; Available in PMC 2026 Oct 15. (PMC7619556; doi:10.1053/j.gastro.2025.02.042)
Supplement: Supplementary file 1 [file EMS217218-supplement-Supplementary_Methods.pdf]

Supplementary Methods

Mouse Lines, Induction of Cre Recombinase In Vivo, Inhibition of Wnt In Vivo

Pdx1-Cre^{ERT}, *LSL-Kras*$^{G12D/+}$, *LSL-Trp53*$^{R172H/+}$, *Rosa26*$^{LSL\text{-}tdRFP}$ mouse lines have all been described previously.[7] Animals were housed in conventional pathogen-free animal facilities and experiments were conducted in accordance with UK Home Office regulations (ASPA 1986 and EU Directive 2010) under the guidelines of Cardiff University Animal Welfare and Ethics Committee. Mice were genotyped by polymerase chain reaction analysis following standard methods and using previously published primer sequences.[7] Both male and female 6- to 8-week-old experimental and control mice were injected with intraperitoneal injection of tamoxifen in corn oil as described previously.[7] Low recombination levels were obtained by administering 1 tamoxifen dose of 1 μg/40 g bodyweight, while high recombination (megadose) was induced by 3 injections of 9 mg/40 g over 5 days.[7] Pancreata were harvested at 7, 35, 70, 140, or 168 days post tamoxifen induction. To test the functional role of Wnt signaling in vivo, 6- to 8-week-old experimental and control mice were induced with low-dose tamoxifen, aged to 35 days post induction of Cre recombinase (p.i.) and treated with WNT-974 1.5 mg/kg or vehicle (DMSO) in corn oil by oral gavage, 5 days per week for 4 weeks. At the end of the treatment pancreatic tissue was harvested and treated as described below. No statistical method was used to predetermine sample size. For most animal studies, experiments were not randomized, and investigators were not blinded to allocation during experiments. To test the functional role of Wnt signaling mice were randomly allocated into vehicle or WNT-974–treated groups. All experiments were reproduced using at least 3 animals of each genotype.

Pancreas Digestion and RNA Sequencing

Pancreas tissues were harvested 35 days post low-dose tamoxifen induction. To reach the minimum of 5000 cells and a minimum of 100 ng RNA/sample for bulk RNA sequencing, we combined 3 pancreas tissues of the same genotype to produce 1 sample per genotype. Each sample was replicated 3 times to generate 3 biological repeats per genotype for sequencing. Pancreas tissues were digested as described previously.[e1] Using an ethylene glycol-bis(β-aminoethyl ether)-*N,N,N′,N′*-tetraacetic acid–based buffer the pancreas from each mouse was inflated and slightly digested with collagenase. The tissue was chopped and further digested using a calcium-based buffer with collagenase. Single-cell digested tissue was stained with leptin PNA-A488 (ThermoFisher) and 4′,6-diamidino-2-phenylindole (ThermoFisher Scientific). 4′,6-Diamidino-2-phenylindole–negative, leptin-positive, RFP-positive cells were sorted using the FACSAria Fusion sorter (BD Biosciences). A minimum of 5000 cells were sorted for each sample. RNA was extracted using the RNeasy Micro kit (Qiagen) following the manufacturer's recommendations. Paired-end sequencing was performed using Sanger sequencing Illumina 1.9. Sequenced reads Fastq files were quality-checked using FastQC. Reads were aligned to the mouse genome (Ensembl-GRCm38 *Mus musculus*) using the STAR package, and reads were counted using the FeatureCounts package, after removing duplicates using the MarkDuplicates tool (GATK). Differential expression was normalized and calculated using DESeq2, comparing the different genotypes against the control. Finally, GSEA software, version 4.2.2 was used for enrichment analysis of different pathways and Prism software, version 10.0.2 (GraphPad) for heatmap graphs and normalized enrichment score graphs. The RNA sequencing data generated during this study are available at GEO ID: GSE255283.

Madin-Darby Canine Kidney Cell Lines and In Vitro Experiments

To assess the interactions between RasV12 and MDCK cells, MDCK-pTR GFP–RasV12 cells were combined with parental MDCK cells at a 1:50 ratio.[9,10] Treatment with tetracycline (2 μg/mL) induces GFP-RasV12 expression in MDCK-pTR GFP–RasV12 cells.[9,10] Cells were plated at a density of 2.5×10^5 cells per well in MW24 plates (Corning) carrying 18-mm diameter glass cover slips (VWR). Mixed cells were incubated for 30 hours or 48 hours at 37°C before fixed with 4% formaldehyde (ThermoFisher Scientific). Cells were then permeabilized and stained (Supplementary Table 14). GFP-RasV12 cells were considered nonextruded when >30% of their cytoplasm was basally protruded. Three technical replicates per experiment in 3 experiments (ie, using 3 independent passages from at least 2 frozen stocks of parental and GFP-RasV12 MDCK cells) were performed and at least 150 GFP-RasV12 cells were quantified per replicate.

For c-Myc silencing, tetracycline-induced GFP-RasV12 cells were transfected with 100 ng siRNA oligos targeting *Myc* using Lipofectamine 3000 (Invitrogen) (siRNA siMyc1: AAGACGUUGUGUGUUCGCCUC – as GAGGCGAACACACAACGUCUU, siRNA siMyc2: AAUUUCAACUGUUCUCGCCGC – as GCGGCGAGAACAGUUGAAAUU and siScr). For cell extrusion and proliferation experiments, GFP-RasV12 cells were trypsinized and mixed with parental MDCK cells 24 hours after siMyc1+2 transfection and were then fixed following a further 48 hours.

For experiments comparing PBS, Wnt3a, and Wnt5a treatments effect, RasV12 cells were mixed 1:50 with parental MDCK cells and once cells were set, induced with tetracycline. Two hours post-tetracycline induction, cells were treated with 1 μg/mL of human recombinant Wnt3a (Peprotech), 100 ng/mL of human recombinant human/mouse Wnt5a (R&D Systems), or PBS (control-Sigma-Aldrich). Cells were fixed 30 hours after PBS, Wnt3a, or Wnt5a treatment for cell extrusion analysis and for analysis of E-cadherin/β-catenin at the RasV12-MDCK contact or within GFP-RasV12 cell clusters. E-cadherin endocytosis and caveolin fluorescence were assessed by fixing cells 16 hours after PBS/Wnt5a treatment.

For Wnt pathway inhibition experiments, tetracycline induced GFP-RasV12 cells were transfected with siMyc1+2/siScr or treated with recombinant Wnt5a/PBS for 24 hours and then mixed with parental MDCK cells. Wnt5a treatment was maintained during the whole experiment. WNT-974 (Stratech) porcupine inhibitor (1 μM) or *OMP*-18R5 (10 mg/mL) was added to the medium 8 hours after GFP-RasV12 and MDCK cells were mixed and plated. DMSO-treated GFP-RasV12 cells were used as control for both inhibitors. To assess cell extrusion, cells were fixed 48 hours after plating. E-cadherin endocytosis was assessed by mixing RasV12 cells with MDCK parental cells, then RasV12 expression was induced by tetracycline and treated with PBS or Wnt5a 2 hours after. Four hours after PBS/Wnt5a treatment, cells were treated with DMSO or WNT-974. Cells were fixed 16 hours after PBS/Wnt5a treatment.

Immunofluorescent staining was performed by permeabilization of cells with 0.05% SDS (Sigma-Aldrich) and 0.05% Triton X100 (Sigma-Aldrich) solution for 15 minutes. Cells were stained with anti–Ki-67, anti–E-cadherin, anti–β-catenin or Caveolin primary antibodies for 2 hours at room temperature, Alexa Fluor secondary antibody incubation for 60 minutes at room temperature and Hoechst (ThermoFisher) (Supplementary Table 14) and mounted using Mowiol (Sigma-Aldrich). For proliferation analysis at least 30 extruded and 30 nonextruded cells were quantified in siMyc 1+2 treated cells per experimental replicate (ie, using 3 independent passages of parental and GFP-RasV12 MDCK cells). For E-cadherin, β-catenin, or Caveolin at least 10 GFP-RasV12 cell clusters per experimental replicate were quantified. Fluorescence intensity at the border between RasV12 and parental MDCK cells were quantified by measuring mean fluorescence intensity of 2.27 $\times$ 2.27-pixel circles (regions of interest) along the membrane separated by approximately 5 pixels. E-cadherin endocytosis was measured quantifying the mean fluorescence intensity of the cytoplasm of each RasV12 cell. At least 10 cells were quantified in 3 technical replicates per experiment in 3 experiments.

Confrontation assays and migration speed analysis were carried out as described.[10] The 1 $\times$ 10^4 cells were plated in inserts (Ibidi) in MW24 plates, allowed to form monolayers (8 hours) before removing the insert and moving the plate to IncuCyte S3 (Sartorius) for live cell imaging. Images were captured every 15 minutes for 48 hours. siMyc/siScr-GFP-RasV12 cells were plated 24 hours after transfection and inserts were removed 8 hours post transfection. For Wnt5a experiments, cells were treated with PBS/Wnt5a for 8 hours before inserts were removed. PBS/Wnt5a treatment was maintained during the whole experiment. WNT-974 porcupine inhibitor (Stratech)/DMSO (Sigma-Aldrich) was added to the medium when inserts were removed. Retraction was measured as the distance GFP-RasV12 cells migrated during 24 hours, following initial cell–cell collision with MDCK cells. Coefficient of boundary smoothness was measured using Fiji in cell confrontation assays at the end of the 24-hour experiment and as described previously.[10] This was calculated by measuring the length of the cell–cell boundary between GFP-RasV12 and MDCK cells at the end of the experiment (*dashed line* in Supplementary Figure 8*A*) divided by the length of a straight line from the top to the bottom of the collision (*solid line* in Supplementary Figure 8*A*). A value of 1.0 indicates a linear boundary. We performed 3 technical replicates per experiment in 3 experiments (ie, using 3 independent passages of parental and GFP-RasV12 MDCK cells).

Western blotting was performed using GFP-RasV12 cell lysates transfected with siMyc/siScr siRNAs at 48 hours post transfection (Supplementary Figure 3*E*), treated for 48 hours with WNT-974/DMSO 24 hours post transfection (Supplementary Figure 5*D*). Cells were lysed using Laemmli buffer (0.0625 M Tris base, 2% sodium dodecyl sulfate, 10% glycerol). Proteins were separated on 12% polyacrylamide gels under reducing polyacrylamide gel electrophoresis conditions. Proteins were then transferred to polyvinylidene difluoride membranes (Immobilon-P, Merck) using wet transfer (1 hour, 100 V). Membranes were blocked in 5% milk Tris-buffered saline with Tween 20 for 1 hour and antibodies were added in the same buffer at the concentrations listed in Supplementary Table 14 for overnight at 4°C or 2 hours at room temperature. Secondary antibodies (Supplementary Table 14) were added in 5% milk Tris-buffered saline with Tween 20 and membranes were developed using ECL luminol kit (Merck) and chemiluminescence films (Amersham Hyperfilm ECL, GE Healthcare). Protein levels were quantified using Fiji (RRID:SCR_002285) software "Gels" tool.

Real-time cell confluency was measured using Incucyte S3 live cell imaging instrument and software (version 2020). The 1 $\times$ 10^3 cells siMyc/siScr-GFP-RasV12 cells were plated 24 hours after transfection with siMyc/siScr siRNAs in MW96 (Corning). Images were captured every 15 minutes for 48 hours. We performed 3 technical replicates per experiment in 3 experiments.

Imaging and Image Analysis

Immunohistochemical imaging was done using the Axio Scan Z1 slide scanner (Zeiss) 20× magnification. Fluorescence imaging of tissue sections was carried out a Zeiss LSM710 confocal microscope. Fluorescence imaging of MDCK/PDEC cell cultures was performed using Zeiss LSM880 or Leica Sp8 confocal microscope.

The percentage of RFP^+ tissue area was determined using endogenous RFP levels quantified as described.[7] Using Fiji (RRID:SCR_002285) software, positive areas were thresholded using RFP fluorescence. Tissue autofluorescence was used to determine total tissue area. The percentage of RFP^+ ducts was analyzed using E-cadherin/RFP/Hoechst-stained sections to identify ducts. At least 20 ducts were quantified per mouse and ducts were considered positive when they contained at least 1 RFP^+ cell. The percentage of RFP^+ cells in pancreatic islets were scored in FFPE immunohistochemical sections stained with anti-RFP antibody and using "positive cell count" tool in QuPath-0.4.1 software. RFP^+ cluster size, perimeter, and area were determined in FFPE immunohistochemical sections stained with anti-RFP antibody and using QuPath software, version 0.4.1 by analyzing shape features of RFP^+ (3,3′-diaminobenzidine tetra hydrochloride–positive) areas. Ki-

67–positive and cleaved caspase-3–positive cells were analyzed using QuPath software, version 0.4.1 positive cell count feature.

Wnt5a fluorescence intensity in the tissue was quantified by masking the tissue area (AF568) and measuring mean fluorescence intensity for Wnt5a (AF488). CD44, β-catenin, and E-cadherin fluorescence intensity at the border between RFP-positive and -negative cells were quantified by measuring mean fluorescence intensity of 2.43 $\times$ 2.43-pixel circles (regions of interest) along the membrane (RFP^+/RFP^-) separated by approximately 5 pixels. CD44, Dvl2, and K-cadherin fluorescence in clusters was quantified by masking RFP^+ (AF568) areas and quantifying mean fluorescence intensity for CD44/Dvl2/K-cadherin (AF488). β-catenin and Sox9 fluorescence in nuclei was quantified by masking nuclei (Hoechst) and quantifying mean fluorescence intensity for β-catenin/Sox9 (AF488) in RFP^+ cells. To avoid noise associated with tissue autofluorescence, fluorescence intensity measurements were made relative to image global mean fluorescence intensity (AF488).

Children's Brain Tumor Network, The Cancer Genome Atlas, and GSE210351 Data Analysis

Children's Brain Tumor Network and The Cancer Genome Atlas data were analyzed using UALCAN data analysis portal. The *z* values represent SDs from the median across samples for the given cancer type. Log2 spectral count ratio values from Clinical Proteomic Tumour Analysis Consortium were first normalized within each sample profile, then normalized across samples. GSE210351 data[45] were analyzed using Geo2R-GEO-NCBI tool.

Statistical Tests

Statistical analyses were performed using Prism 10 software (GraphPad). Normally distributed data, as determined by the Shapiro-Wilke test or D'Agostino and Pearson test were analyzed using unpaired Student *t* tests. Human data statistical analysis was performed using the UALCAN data analysis portal. A *P* value of $<.05$ was considered as significant and a rejection of the null hypothesis. Graphical data represent mean $\pm$ SD. No statistical method was used to predetermine sample size. Definition of *n* is in the figures. GSEA was performed using GSEA software, version 4.2.2. GSEA was used to identify gene sets that were differentially expressed between 2 groups of samples. Gene sets were considered enriched if they had a false discovery rate of <0.25. Heatmaps were created by computing normalized gene counts for each individual sample into row *z* scores. SuperPlots show all the quantified points (smaller circles) and the mean for each mouse/experiment (larger circles). Source data for all figures can be found in Supplementary Table 13.

Supplementary References

e1. Assi M, Dauguet N, Jacquemin P. DIE-RNA: a reproducible strategy for the digestion of normal and injured pancreas, isolation of pancreatic cells from genetically engineered mouse models and extraction of high quality RNA. Front Physiol 2018;9:129.

e2. Dudgeon C, Harris CR, Chen Y, et al. A novel model of pancreatic cancer dormancy reveals mechanistic insights and a dormancy gene signature with human relevance. 2020. bioRxiv 2020;04.13:037374.

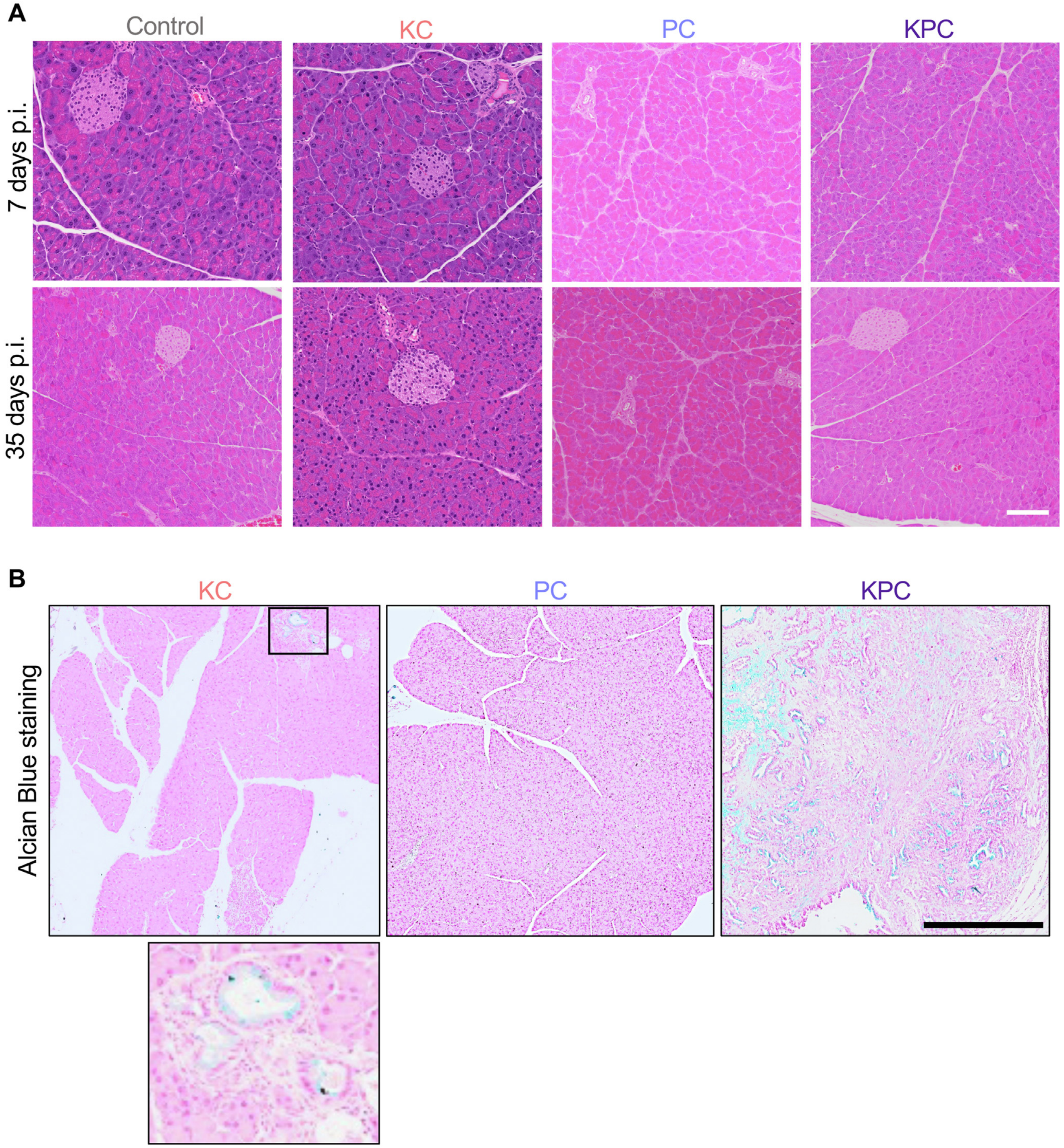

Supplementary Figure 1. Noneliminated mutant cells can progress to mucin-positive lesions. (*A*) H&E staining of pancreas tissues harvested at 35 days p.i. from control, KrasG12D (KC), p53R172H (PC) or double mutant (KPC) expressing mice. *Scale bar:* 100 μm. (*B*) Pancreas tissues from KC, PC, and KPC mice harvested at 168 days p.i. and stained for mucin (using Alcian blue). *Scale bar:* 500 μm. *Boxed area* shown magnified (*lower panel*).

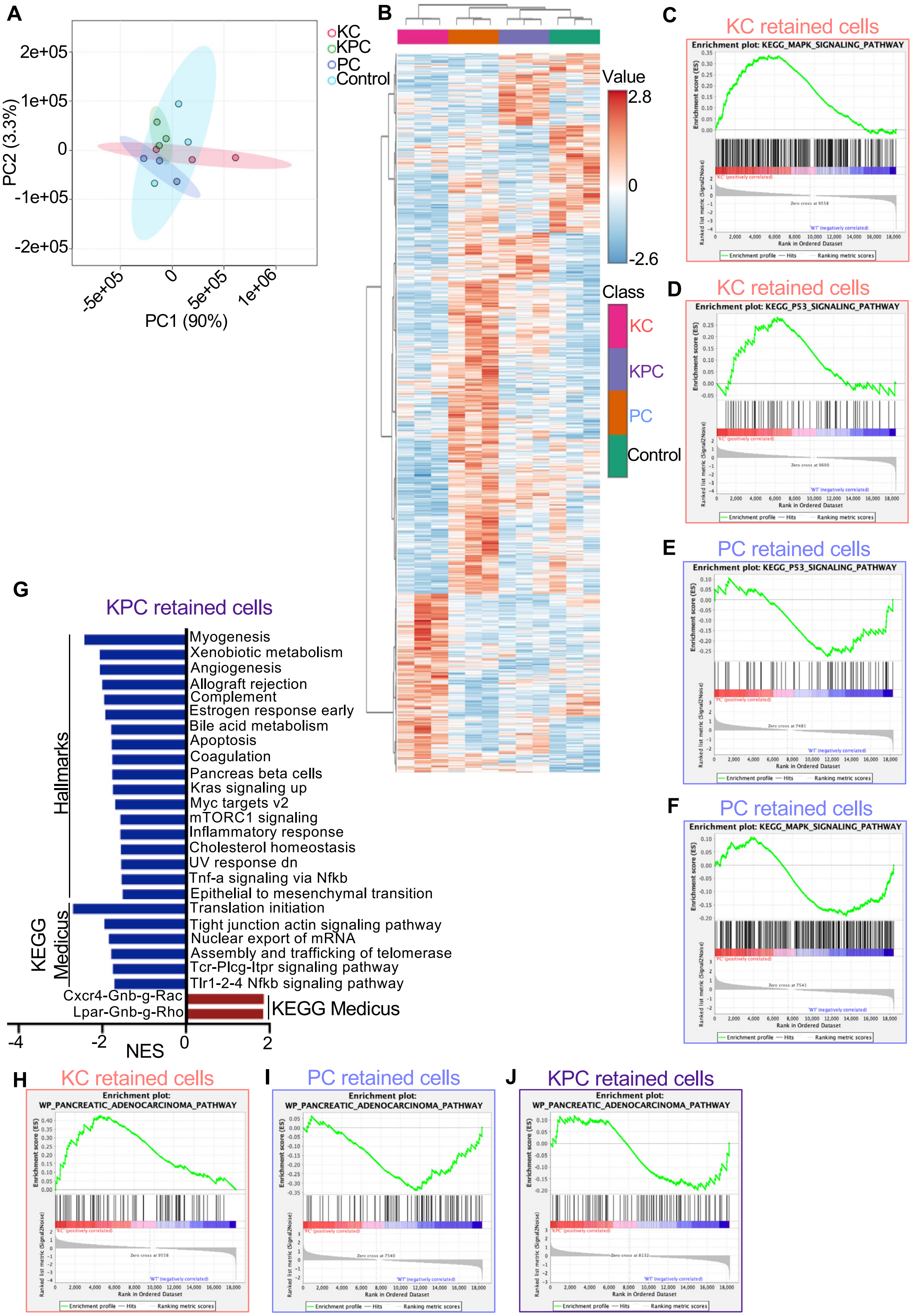
A
B
C
D
E
F
G
H
I
J
KC
KPC
PC
Control
PC2 (3.3%)
PC1 (90%)
Value
Class
KC retained cells
PC retained cells
KPC retained cells
Enrichment plot: KEGG_MAPK_SIGNALING_PATHWAY
Enrichment plot: KEGG_P53_SIGNALING_PATHWAY
Enrichment plot: WP_PANCREATIC_ADENOCARCINOMA_PATHWAY
Hallmarks
KEGG Medicus
Myogenesis
Xenobiotic metabolism
Angiogenesis
Allograft rejection
Complement
Estrogen response early
Bile acid metabolism
Apoptosis
Coagulation
Pancreas beta cells
Kras signaling up
Myc targets v2
mTORC1 signaling
Inflammatory response
Cholesterol homeostasis
UV response dn
Tnf-a signaling via Nfkb
Epithelial to mesenchymal transition
Translation initiation
Tight junction actin signaling pathway
Nuclear export of mRNA
Assembly and trafficking of telomerase
Tcr-Plcg-Itpr signaling pathway
Tlr1-2-4 Nfkb signaling pathway
Cxcr4-Gnb-g-Rac
Lpar-Gnb-g-Rho
NES

Supplementary Figure 2. Transcriptomic analysis indicates Kras and p53 pathways deregulation in retained cells. (*A*) Principal component analysis of control, KrasG12D (KC), p53R172H (PC) or double-mutant (KPC) transcriptomic data obtained at 35 days p.i. PC1 and PC2 of normalized data are shown. (*B*) Unsupervised clustering heatmap of the top 2500 differentially expressed genes (after normalization) between the 12 samples sequenced (3× Control, KC, PC and KPC). GSEA enrichment plots showing (*C*) KEGG_MAPK_signaling_pathway (M10792) and (*D*) KEGG_p53_signaling_pathway (M6370) positive correlation in KC cells compared to control and (*E*) KEGG_p53_signaling_pathway (M6370), and (*F*) KEGG_MAPK_signaling_pathway (M10792) negative correlation in PC cells compared to control. (*G*) Normalized enrichment scores (NES) of GSEA on the Hallmarks and KEGG Medicus gene sets for the RNA sequencing experiment analysis of KPC retained cells. Only gene sets with an false discovery rate of $<$0.25 were included in the *graph*. The complete list that contains the results of GSEA analysis is provided in Supplementary Tables 8–9. GSEA enrichment plots of WP_pancreatic_adenocarcinoma_pathway (M39732) positively correlated in (*H*) KC cells and negatively correlated in (*I*) PC cells and (*J*) KPC cells compared to control.

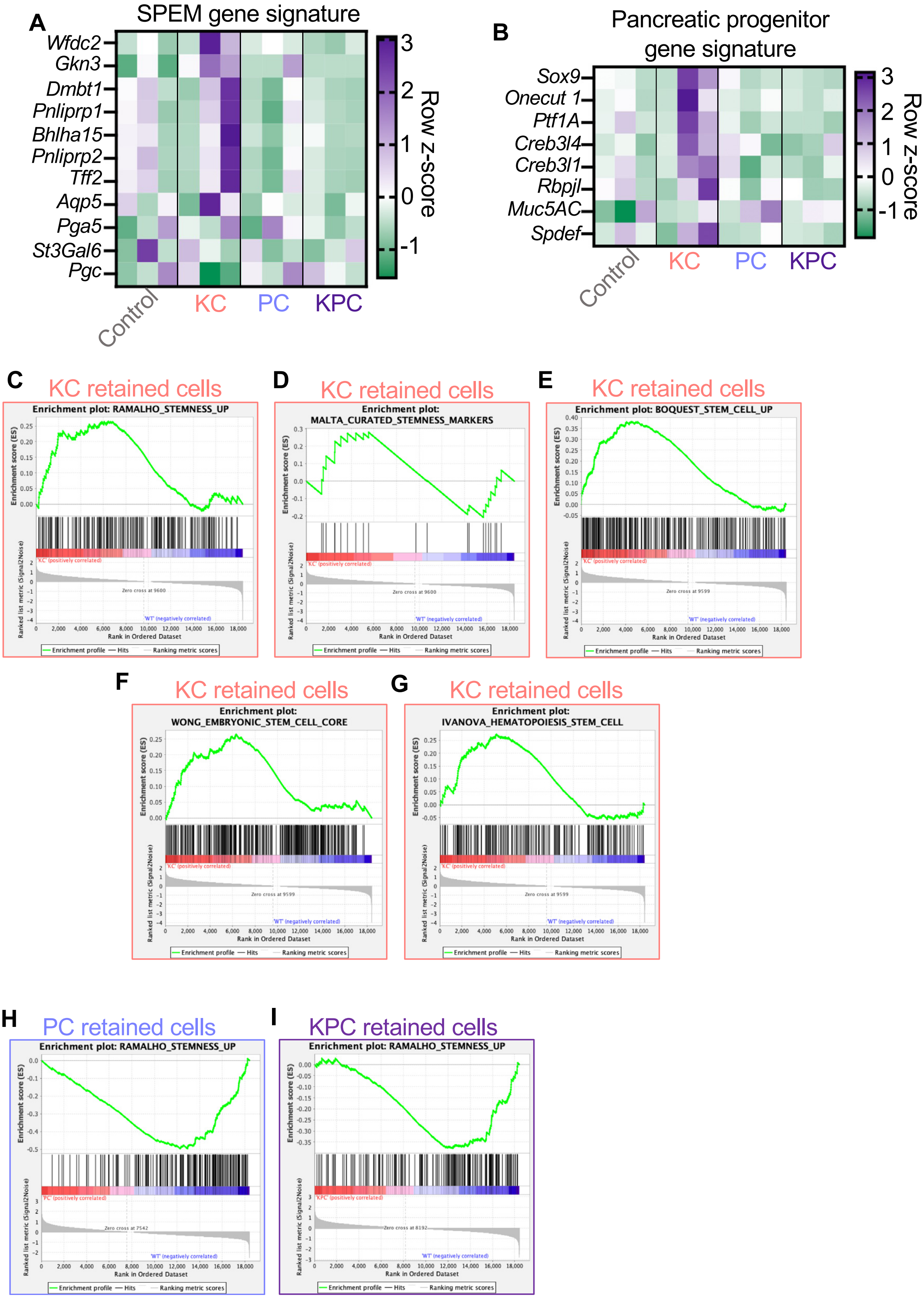
A
SPEM gene signature
Wfdc2
Gkn3
Dmbt1
Pnliprp1
Bhlha15
Pnliprp2
Tff2
Aqp5
Pga5
St3Gal6
Pgc
Control
KC
PC
KPC
Row z-score
B
Pancreatic progenitor gene signature
Sox9
Onecut 1
Ptf1A
Creb3l4
Creb3l1
Rbpjl
Muc5AC
Spdef
Control
KC
PC
KPC
Row z-score
C
KC retained cells
Enrichment plot: RAMALHO_STEMNESS_UP
D
KC retained cells
Enrichment plot: MALTA_CURATED_STEMNESS_MARKERS
E
KC retained cells
Enrichment plot: BOQUEST_STEM_CELL_UP
F
KC retained cells
Enrichment plot: WONG_EMBRYONIC_STEM_CELL_CORE
G
KC retained cells
Enrichment plot: IVANOVA_HEMATOPOIESIS_STEM_CELL
H
PC retained cells
Enrichment plot: RAMALHO_STEMNESS_UP
I
KPC retained cells
Enrichment plot: RAMALHO_STEMNESS_UP
Enrichment score (ES)
Ranked list metric (Signal2Noise)
Rank in Ordered Dataset
Enrichment profile
Hits
Ranking metric scores

Supplementary Figure 3. Noneliminated KrasG12D (KC) are stem-like, whereas p53R172H-expressing populations are not. (*A, B*) *Heatmaps* showing gene expression in noneliminated KrasG12D (KC) cells and control of (*A*) spasmolytic polypeptide-expressing metaplasia and (*B*) pancreatic lineage. *Heatmaps* show row *z* scores for the expression of each gene for 3 samples (3 pooled mice per sample) per genotype obtained from the RNA sequencing experiment. Genes are listed in *rows*, genotypes in *columns*. GSEA enrichment plots indicate positive correlation in KC retained cells compared to control for (*C*) Enrichment of Ramalho_stemness_up (M9473), (*D*) Malta_curated_stemness_markers (M30411) (embryonic, neural, and hematopoietic stem cells), (*E*) Boquest_stem_cell_up (M1834), (*F*) Wong_embryonic_stem_cell_core (M7079), and (*G*) Ivannova_hematopoiesis_-stem_cell (M6813). GSEA enrichment plots indicate negative correlation for Ramalho_Stemness_up (M30411) in (*H*) p53R172H (PC) retained cells and (*I*) KrasG12D p53R172H (KPC) retained cells compared to control. For more information on source data see Supplementary Table 13.

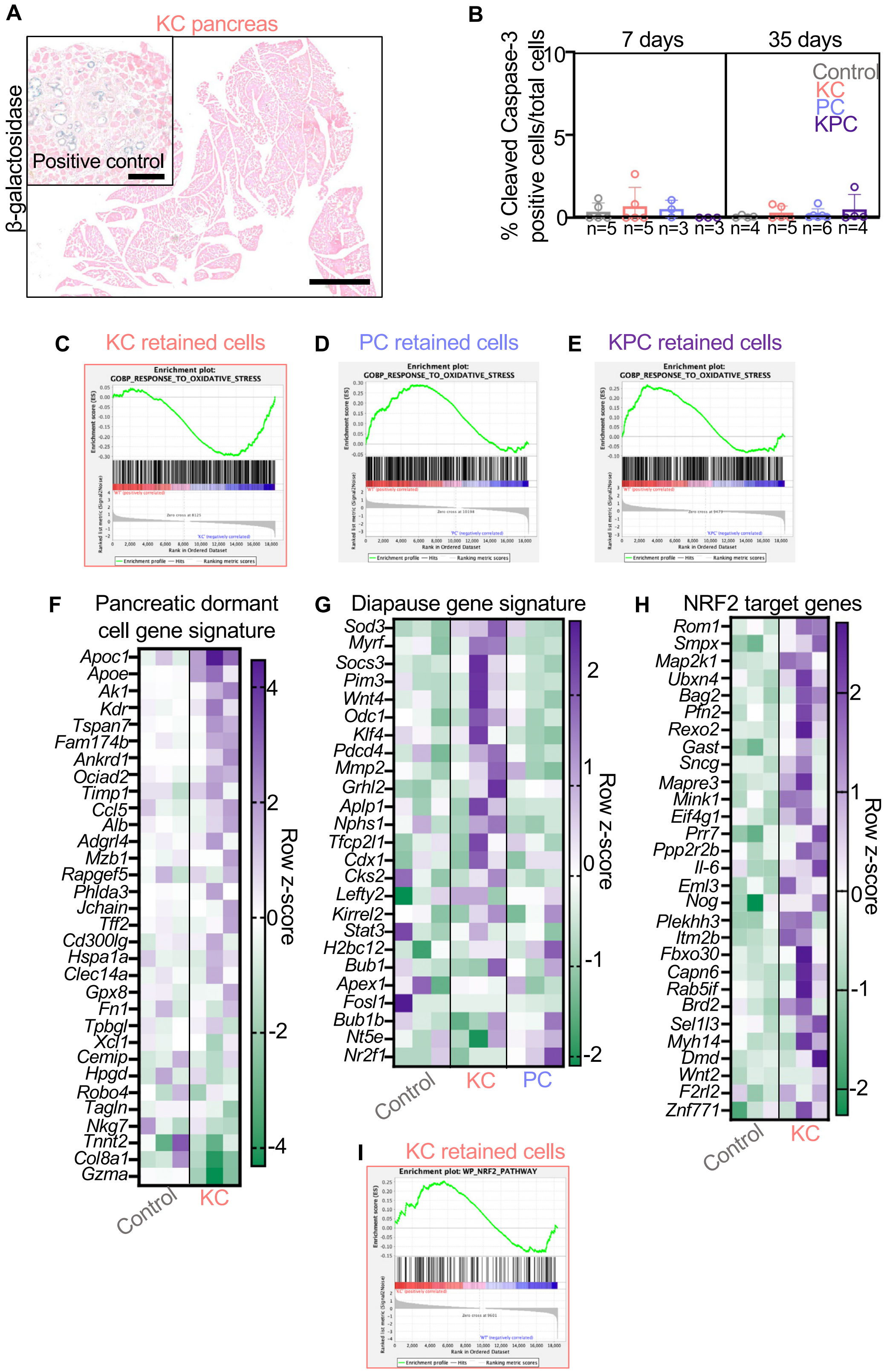
A
KC pancreas
β-galactosidase
Positive control
B
7 days
35 days
% Cleaved Caspase-3 positive cells/total cells
Control
KC
PC
KPC
n=5 n=5 n=3 n=3 n=4 n=5 n=6 n=4
C
KC retained cells
D
PC retained cells
E
KPC retained cells
Enrichment plot: GOBP_RESPONSE_TO_OXIDATIVE_STRESS
F
Pancreatic dormant cell gene signature
Apoc1
Apoe
Ak1
Kdr
Tspan7
Fam174b
Ankrd1
Ociad2
Timp1
Ccl5
Alb
Adgrl4
Mzb1
Rapgef5
Phlda3
Jchain
Tff2
Cd300lg
Hspa1a
Clec14a
Gpx8
Fn1
Tpbgl
Xcl1
Cemip
Hpgd
Robo4
Tagln
Nkg7
Tnnt2
Col8a1
Gzma
Control
KC
Row z-score
G
Diapause gene signature
Sod3
Myrf
Socs3
Pim3
Wnt4
Odc1
Klf4
Pdcd4
Mmp2
Grhl2
Aplp1
Nphs1
Tfcp2l1
Cdx1
Cks2
Lefty2
Kirrel2
Stat3
H2bc12
Bub1
Apex1
Fosl1
Bub1b
Nt5e
Nr2f1
Control
KC
PC
Row z-score
H
NRF2 target genes
Rom1
Smpx
Map2k1
Ubxn4
Bag2
Pfn2
Rexo2
Gast
Sncg
Mapre3
Mink1
Eif4g1
Prr7
Ppp2r2b
Il-6
Eml3
Nog
Plekhh3
Itm2b
Fbxo30
Capn6
Rab5if
Brd2
Sel1l3
Myh14
Dmd
Wnt2
F2rl2
Znf771
Control
KC
Row z-score
I
KC retained cells
Enrichment plot: WP_NRF2_PATHWAY

Supplementary Figure 4. Noneliminated KrasG12D cells up-regulate cell dormancy-associated pathways. (*A*) Representative *images* of β-galactosidase staining of pancreas tissue harvested from a KrasG12D (KC) mouse at 35 days p.i. (*scale bar:* 1000 μm) and in PanIN lesions as positive control (*scale bar:* 250 μm). (*B*) Percentage of cleaved caspase-3–positive cells/total cells in control, KC, p53R172H (PC) or double-mutant (KPC) pancreas tissues harvested at 7 and 35 days p.i. mean $\pm$ SD per mouse. N $=$ number of mice described in the *graph*. (*C, D, E*) GSEA enrichment plots for GOBP_RESPONSE_TO_OXIDATIVE_STRESS (M3223) showing (*C*) negative correlation in KC retained cell signatures compared to control; positive correlation for (*D*) PC and (*E*) KPC retained cell signatures compared to control. *Heatmaps* showing gene expression in (*F*) pancreatic dormant cell gene signature[e2] (*G*) diapause gene expression[22] and (*H*) NRF2 target genes (NRF2_Q4 M14141) (*rows*) in control, KC or p53R172H (PC) cell transcriptomes (columns). The *heatmap* shows row *z* scores for each gene obtained from 3 samples per genotype (3 pooled mice per samples) from the RNA sequencing experiment. Only genes with *z* scores of a fold change of 2 (*F*) or 2.25 (*H*) are shown. The full list of genes can be found in Supplementary Tables 11 and 13. (*I*) GSEA enrichment plots showing positive correlation in KC retained cells compared to control for WP_NRF2_pathway (M39454).

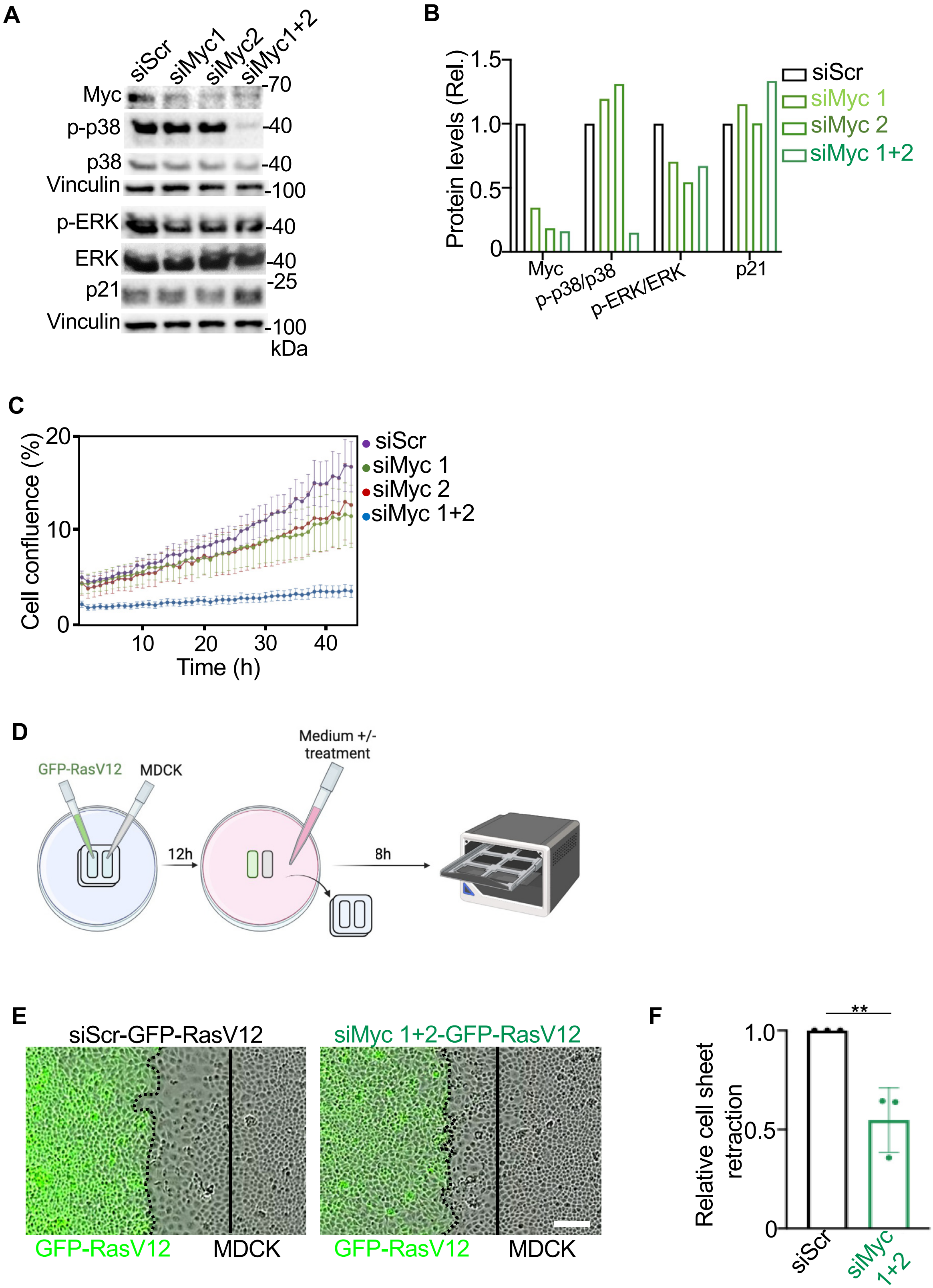
A
siScr
siMyc1
siMyc2
siMyc1+2
Myc
p-p38
p38
Vinculin
p-ERK
ERK
p21
Vinculin
-70
-40
-40
-100
-40
-40
-25
-100
kDa
B
Protein levels (Rel.)
1.5
1.0
0.5
0
Myc
p-p38/p38
p-ERK/ERK
p21
siScr
siMyc 1
siMyc 2
siMyc 1+2
C
Cell confluence (%)
20
10
0
10
20
30
40
Time (h)
siScr
siMyc 1
siMyc 2
siMyc 1+2
D
GFP-RasV12
MDCK
12h
Medium +/- treatment
8h
E
siScr-GFP-RasV12
siMyc 1+2-GFP-RasV12
GFP-RasV12
MDCK
GFP-RasV12
MDCK
F
Relative cell sheet retraction
1.0
0.5
0
**
siScr
siMyc 1+2

Supplementary Figure 5. Cell cycle arrest abrogates RasV12 cell extrusion in vitro. (*A*) Immunodetection of indicated antigens in GFP-RasV12 cells transfected with scrambled siRNA (siScr) or 2 siRNA oligos targeting endogenous *Myc* (siMyc1, siMyc2 or combined siMyc1+2). Lysates were collected 48 hours after transfection. p-p38, phospho-p38; p-ERK, phospho-ERK. Anti-vinculin staining was included as protein loading control. (*B*) Quantification of protein levels in the blot in (*A*). Values are relative to siScr protein levels. (*C*) Real-time cell confluence of GFP-RasV12 cells transfected with different siRNAs as quantified via Incucyte S3 imaging. Cell confluence was determined in cells 12 hours post siRNA transfection over 48 hours. Data indicate mean $\pm$ SD for 3 experiments per condition. (*D*) Schematic representation of cell confrontation assay experiments. Illustration created with BioRender.com. (*E*) Representative time-lapse images of cell confrontation assays. GFP-RasV12 cells expressing either siScr or siMyc1+2 confront nonlabeled parental MDCK cells. *Dashed lines* highlight the border between GFP-RasV12 cells and MDCK cells. *Solid lines* indicate MDCK cell migration front at the beginning of the experiment. *Scale bar:* 100 μm. (*F*) Relative retraction distance during 24 hours of siScr-GFP-RasV12 (*black bar*) or combined Myc1+2 siRNA (*green bar*) after collision with parental MDCK. Results are presented as retraction distance in siMyc-GFP-RasV12 relative to siScr-GFP-RasV12. Data represent mean $\pm$ SD measurements of 3 experiments. Student *t* test was used to analyze the data. ***P* $<$ .005.

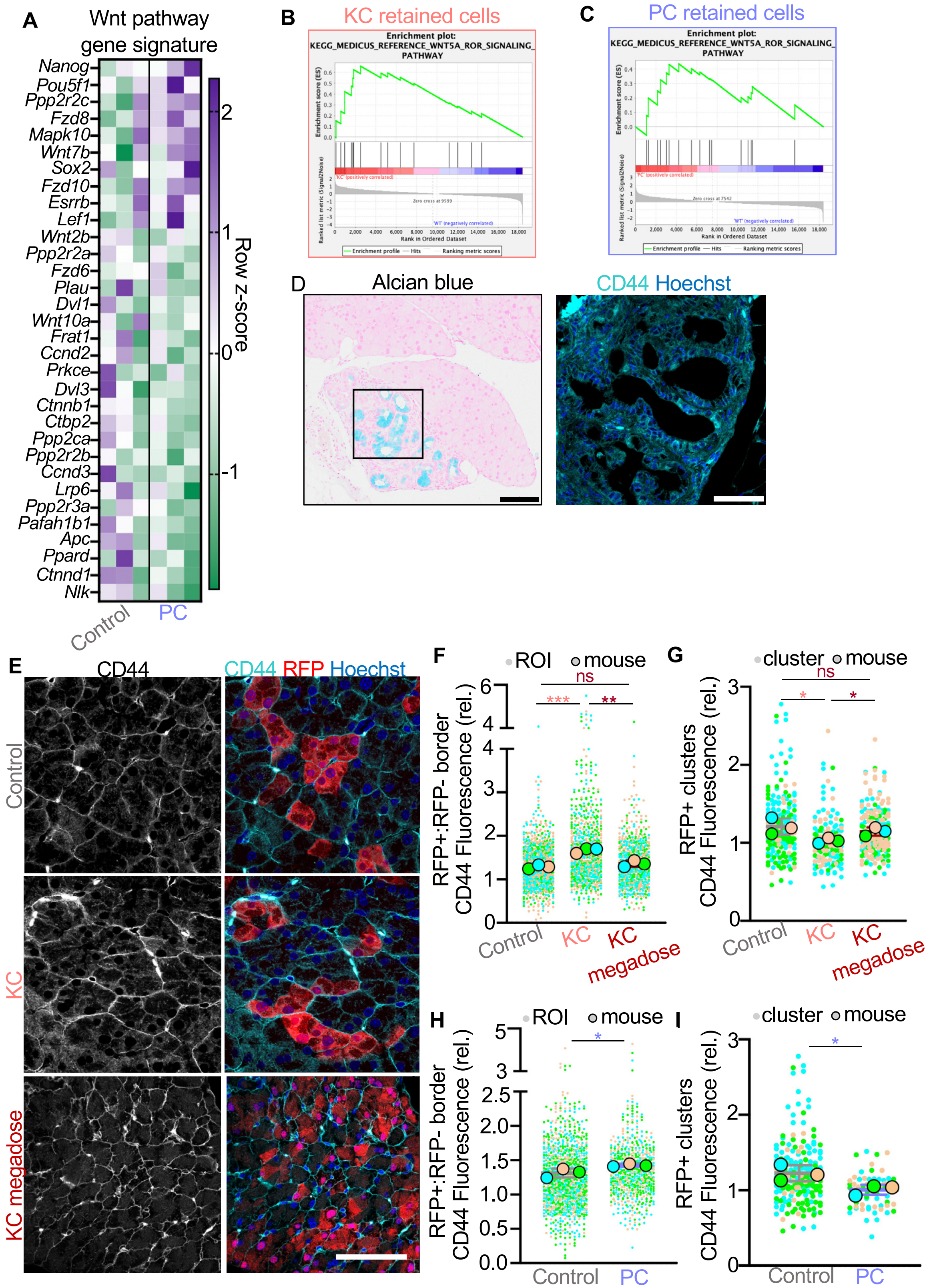
A
Wnt pathway gene signature
Nanog
Pou5f1
Ppp2r2c
Fzd8
Mapk10
Wnt7b
Sox2
Fzd10
Esrrb
Lef1
Wnt2b
Ppp2r2a
Fzd6
Plau
Dvl1
Wnt10a
Frat1
Ccnd2
Prkce
Dvl3
Ctnnb1
Ctbp2
Ppp2ca
Ppp2r2b
Ccnd3
Lrp6
Ppp2r3a
Pafah1b1
Apc
Ppard
Ctnnd1
Nlk
Row z-score
Control
PC
B
KC retained cells
Enrichment plot: KEGG_MEDICUS_REFERENCE_WNT5A_ROR_SIGNALING_PATHWAY
C
PC retained cells
Enrichment plot: KEGG_MEDICUS_REFERENCE_WNT5A_ROR_SIGNALING_PATHWAY
D
Alcian blue
CD44 Hoechst
E
CD44
CD44 RFP Hoechst
Control
KC
KC megadose
F
ROI
mouse
RFP+:RFP- border CD44 Fluorescence (rel.)
ns
***
**
Control
KC
KC megadose
G
cluster
mouse
RFP+ clusters CD44 Fluorescence (rel.)
ns
*
*
Control
KC
KC megadose
H
ROI
mouse
RFP+:RFP- border CD44 Fluorescence (rel.)
*
Control
PC
I
cluster
mouse
RFP+ clusters CD44 Fluorescence (rel.)
*
Control
PC

Supplementary Figure 6. Wnt pathway is active in noneliminated KrasG12D or p53R172H cells. (*A*) *Heatmap* of Wnt signaling pathway (WP_Wnt_signaling, MM15829) related genes (rows) in Control and p53R712H (PC) cell transcriptomes (*columns*). The *heatmap* shows row *z* scores for each gene obtained from the RNA sequencing experiment. Only genes with *z* scores of a fold-change of 2 are shown in the Wnt pathway panel. The full list of genes can be found in Supplementary Table 12. GSEA enrichment analysis plots showing positive correlation in (*B*) KrasG12D (KC) and (*C*) PC retained cells compared with control for the KEGG_MEDICUS_reference_WNT5A_ROR_signaling_pathway (M47822). (*D*) Representative *images* of PanINs in pancreas tissue sections harvested from KC mice at 140 days p.i. *Left:* Mucin-positive PanINs (*blue*). *Scale bar:* 100 μm. *Right:* Confocal image of anti-CD44 antibody (*cyan*) labeling and Hoechst (*blue*) in PanINs in a consecutive tissue slice. *Scale bar:* 50 μm. (*E*) Representative confocal *images* of pancreas tissues harvested at 35 days p.i. stained with CD44 and RFP from Control, KC and KrasG12D high-dose tamoxifen (9 mg/40 g over 5 days, KC megadose) mice. *Scale bar:* 50 μm. (*F–I*) Mean fluorescence intensity relative to background of CD44 at the boundary between RFP-positive and RFP-negative cells (*F, H*) or in entire RFP-positive clusters (*G, I*) in control, KrasG12D low-dose (1 mg/40 g, KC) and KrasG12D high-dose tamoxifen (KC megadose) tissues (*F, G*) or control and PC tissues (*H, I*) harvested at 35 days p.i. CD44 fluorescence was quantified and reported as in Figure 4*H*. *SuperPlot* shows all the quantified regions of interest (ROI) (*F, H*) or RFP-positive clusters (*G, I*) (*smaller circles*) and the mean for each mouse (n = 3 samples; *larger circles*). The graphs show mean ± SD for the 3 mice. **P* < .05;***P* < .001; ****P* < .0001. ns, *P* > .05 (Student *t* test).

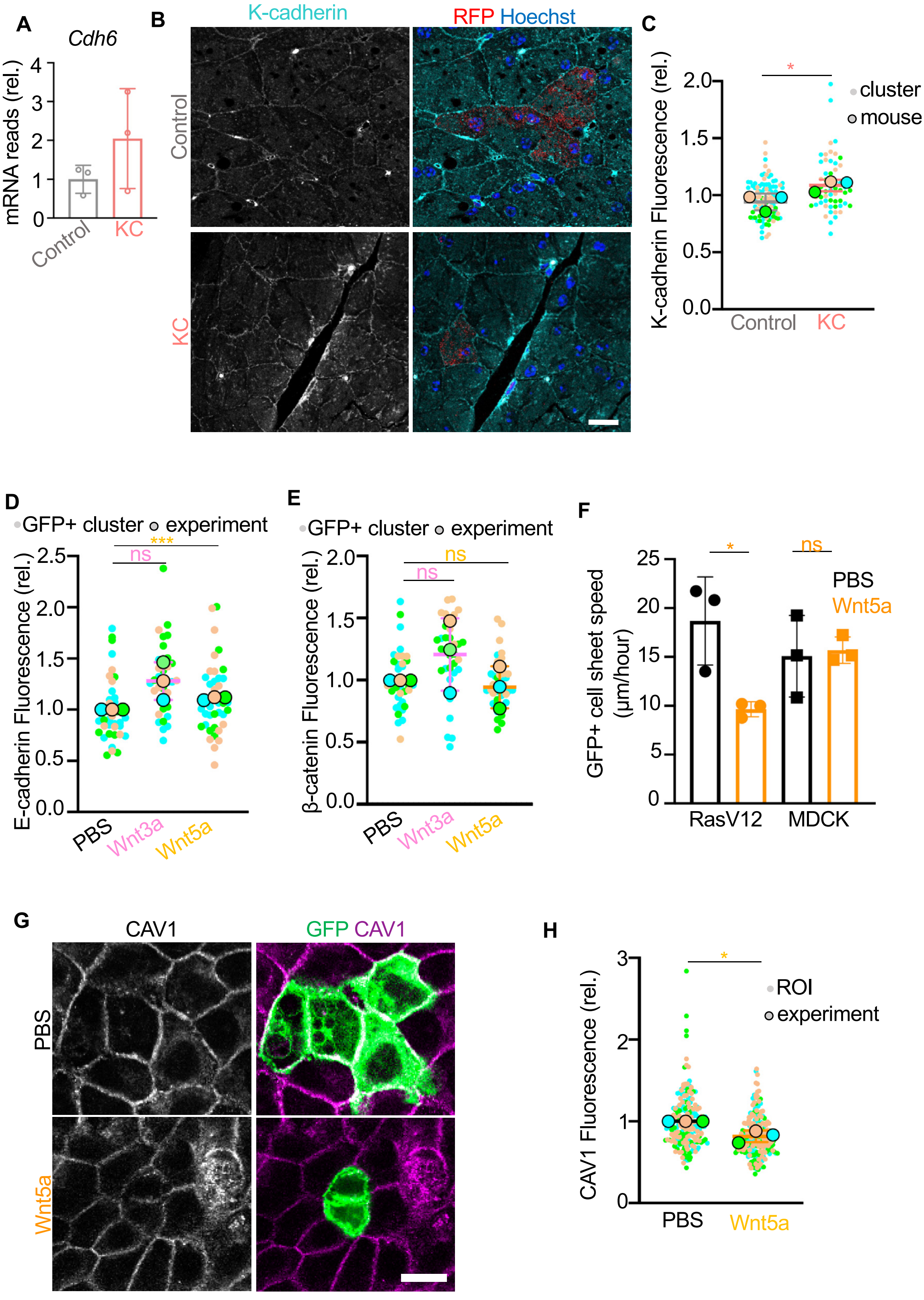
A
Cdh6
mRNA reads (rel.)
Control
KC
B
K-cadherin
RFP Hoechst
Control
KC
C
K-cadherin Fluorescence (rel.)
cluster
mouse
Control
KC
D
GFP+ cluster experiment
E-cadherin Fluorescence (rel.)
ns
***
PBS
Wnt3a
Wnt5a
E
GFP+ cluster experiment
β-catenin Fluorescence (rel.)
ns
PBS
Wnt3a
Wnt5a
F
GFP+ cell sheet speed (μm/hour)
PBS
Wnt5a
RasV12
MDCK
G
CAV1
GFP CAV1
PBS
Wnt5a
H
CAV1 Fluorescence (rel.)
ROI
experiment
PBS
Wnt5a

Supplementary Figure 7. Wnt5a increases RasV12 cell cohesion. (*A*) *Cdh6* messenger RNA reads relative to control reads in the 3 samples in control and 3 samples in KrasG12D (KC) obtained from the RNA sequencing experiment. *Graph* represents mean ± SD reads. (*B*) Representative confocal *images* of pancreas tissues stained with K-cadherin and RFP harvested at 35 days p.i. from control and KC mice. *Scale bar:* 50 μm. (*C*) Mean fluorescence intensity relative to background of K-cadherin RFP-positive clusters in control or KC pancreas tissues harvested at 35 days p.i. *SuperPlot* shows all the quantified RFP^+ clusters (*smaller circles*) and the mean for each mouse (n = 3 samples; *larger circles*). The *graph* shows mean ± SD for the 3 mice. *P = .0474 (Student *t* test). (*D, E, H*) Mean fluorescence intensity of E-cadherin (*D*), β-catenin (*E*), or Caveolin-1 (CAV1) (*H*) in GFP-RasV12 cell clusters (mixed with MDCK cells) treated with PBS (*black*), Wnt3a (*pink*), or Wnt5a (*yellow*) for 30 hours (*D, E*) or 16 hours (*H*) relative to PBS-treated cells. *SuperPlot* shows all the quantified GFP-RasV12 clusters (*smaller circles*) and the mean for each experiment (n = 3 samples; *larger circles*). The *graphs* show mean ± SD for the 3 experiments. *P = .0117; ***P = .0003; ns, $P > .05$ (Student *t* test). (*F*) Cell speed (μm/h) at which RasV12 (*circles*) or MDCK (*squares*) cells treated with PBS (*black*) or Wnt5a (*yellow*) migrate. The data represent mean ± SD from 3 independent experiment. *P = .0268; ns, P = .8202 (Student *t* test). (*G*) Confocal *images* of CAV1 in GFP-RasV12 cells mixed with MDCK cells treated with PBS or Wnt5a for 16 hours. *Scale bar:* 20 μm.

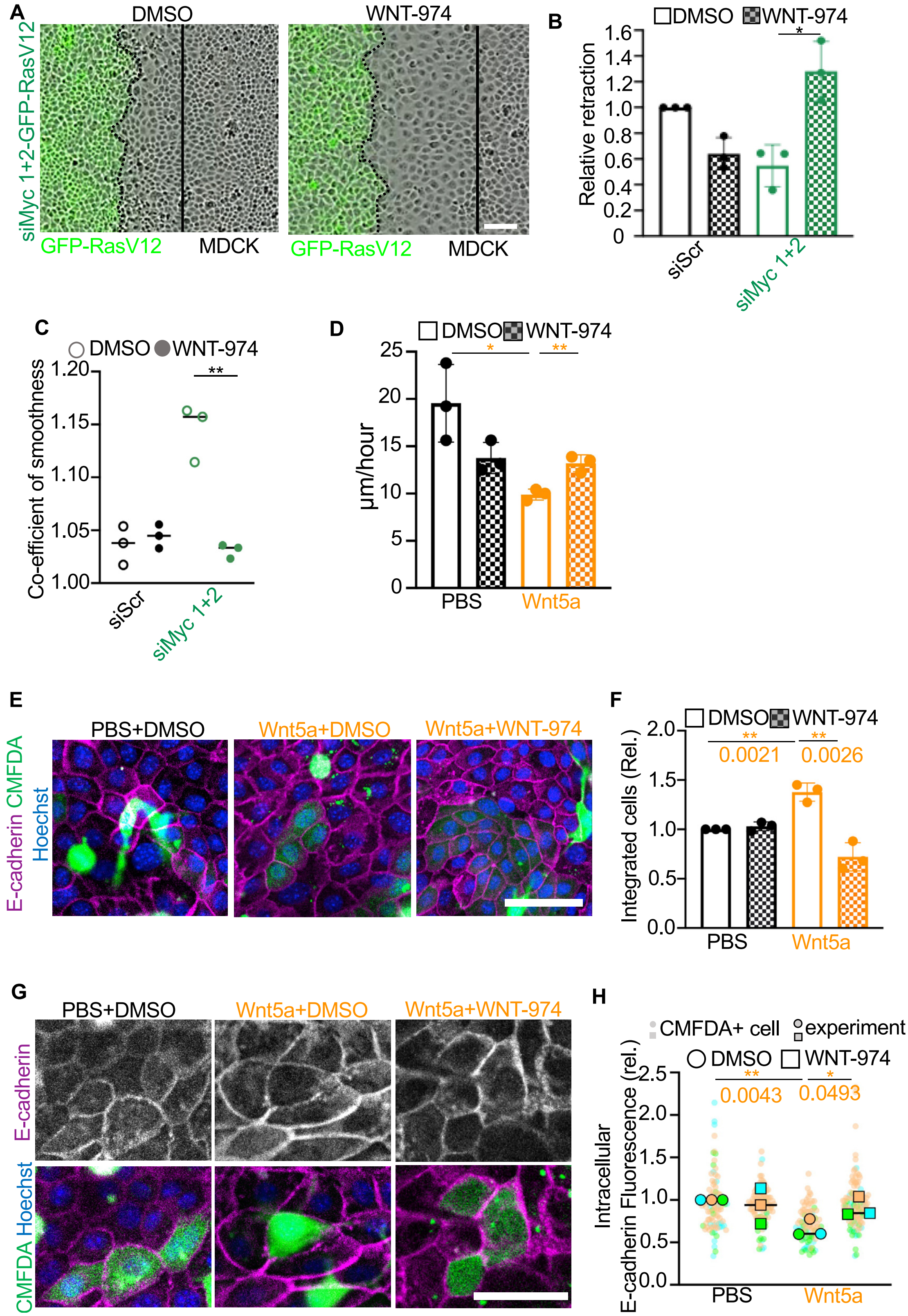
A
DMSO
WNT-974
siMyc 1+2-GFP-RasV12
GFP-RasV12
MDCK
B
DMSO
WNT-974
Relative retraction
siScr
siMyc 1+2
C
DMSO
WNT-974
Co-efficient of smoothness
siScr
siMyc 1+2
D
DMSO
WNT-974
µm/hour
PBS
Wnt5a
E
PBS+DMSO
Wnt5a+DMSO
Wnt5a+WNT-974
E-cadherin CMFDA
Hoechst
F
DMSO
WNT-974
0.0021
0.0026
Integrated cells (Rel.)
PBS
Wnt5a
G
PBS+DMSO
Wnt5a+DMSO
Wnt5a+WNT-974
E-cadherin
CMFDA Hoechst
H
CMFDA+ cell
experiment
DMSO
WNT-974
0.0043
0.0493
Intracellular E-cadherin Fluorescence (rel.)
PBS
Wnt5a

Supplementary Figure 8. Cell cycle arrest and Wnt signaling prevent GFP-RasV12/KC-TDEC segregation from normal MDCK/PDEC cells in vitro. (*A*) Representative time-lapse images of cell confrontation assays. GFP-RasV12 cells expressing combined Myc1+2 siRNA (siMyc 1+2-GFP-RasV12) confront nonlabeled parental MDCK cells. Assays were conducted in the presence of DMSO or WNT-974 (1 mM). *Dashed lines* highlight the border between GFP-RasV12 cells and MDCK cells. *Solid lines* indicate migration front of MDCK cells at the beginning of the experiment. *Scale bar:* 100 μm. (*B*) Retraction distance of GFP-RasV12 cells transfected with siScr; *black bars*) or siMyc-GFP-RasV12; *green bars*) after collision with parental MDCK cells until 24 hours later. Assays were conducted in the presence of DMSO (*solid bars*) or WNT-974 (*hatched bars*). Values are relative to PBS-/DMSO-treated cells. Data represent mean ± SD of 3 experiments. **P* < .05 (Student *t* test). (*C*) Coefficient of boundary smoothness in cell confrontation assay. Data represent mean ± SD of 3 experiments. ***P* < .005 (Student *t* test). (*D*) Cell speed (μm/h) at which RasV12 cells treated with PBS (*black*) or Wnt5a (*yellow*) and DMSO or WNT-974 migrate. The data represent mean ± SD from 3 independent experiment. **P* = .0156; ***P* = .0058 (Student *t* test). (*E, G)* Confocal *images* of transformed KrasG12D ductal epithelial cells (*green*) mixed with nontransformed PDECs at 1:50 ratios in vitro showing integrated/nonintegrated KrasG12D cells (*E*) or E-cadherin intracellular levels (*G*). Cells were pretreated with PBS (*black*) or Wnt5a (*orange*) for 6 hours and fixed 48 hours after mixing and DMSO or WNT-974 treatment. *Scale bar:* 50 μm. (*F*) Proportion of integrated KrasG12D ductal epithelial cells relative to integrated PBS-/DMSO-treated KrasG12D ductal epithelial cells (n = 3 experiments). Cells were treated as in E. Data represent mean ± SD. Student *t* test was used to analyze the data. ***P* < .001. (*H*) Mean fluorescence intensity of intracellular E-cadherin in KrasG12D ductal epithelial cells relative to PBS-/DMSO-treated cells (n = 3 experiments). Cells were treated as in (*E*). Data represent mean ± SD. Student *t* test was used to analyze the data. **P* < .05; ***P* < .001.

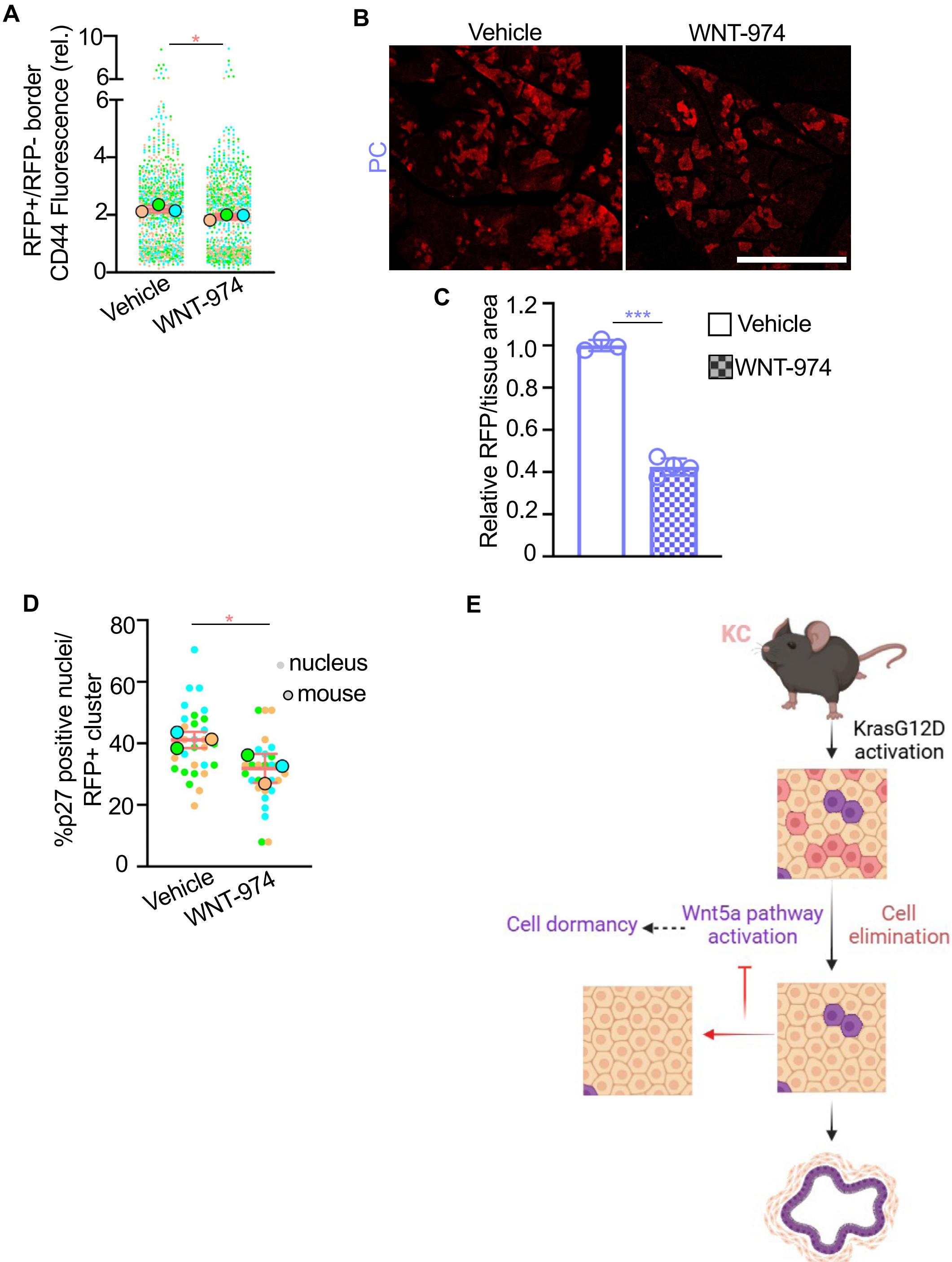

Supplementary Figure 9. Wnt signaling inhibition promotes KrasG12D (KC) cell elimination in vivo. (*A*) Mean fluorescence intensity relative to background of CD44 at the boundary between RFP-positive and RFP-negative cells in vehicle or WNT-974–treated KrasG12D (1.5 mg/kg, KC) tissues harvested at the end of the treatment (see Figure 6*H*). CD44 fluorescence was quantified and reported as in Figure 4*H*. Control quantification is the same used in Supplementary Figure 6*F*. *SuperPlot* shows all the quantified regions of interest (ROIs) (*smaller circles*) and the mean for each mouse (n = 3 samples; *larger circles*). The *graph* shows mean ± SD for the 3 mice. Student *t* test was used to analyze the data. $*P = .0467$. (*B*) Representative *images* of endogenous RFP fluorescence in PC pancreas tissue sections harvested 28 days post treatment with vehicle or WNT-974 (1.5 mg/kg). *Scale bar:* 500 μm. (*C*) Percentage of RFP area in PC mice treated with WNT-974 or vehicle and relative to vehicle-treated tissues. Data represent mean ± SD per mouse. Student *t* test was used to analyze the data, $***P < .0005$. (*D*) Percentage of p27-positive nuclei in RFP^+ clusters from KC tissues treated with vehicle or WNT-974. Data represent mean ± SD per mouse. Student *t* test was used to analyze the data, $*P = .0410$. (*E*) Schematic summarizing the main findings of the study. Illustration created with BioRender.com.

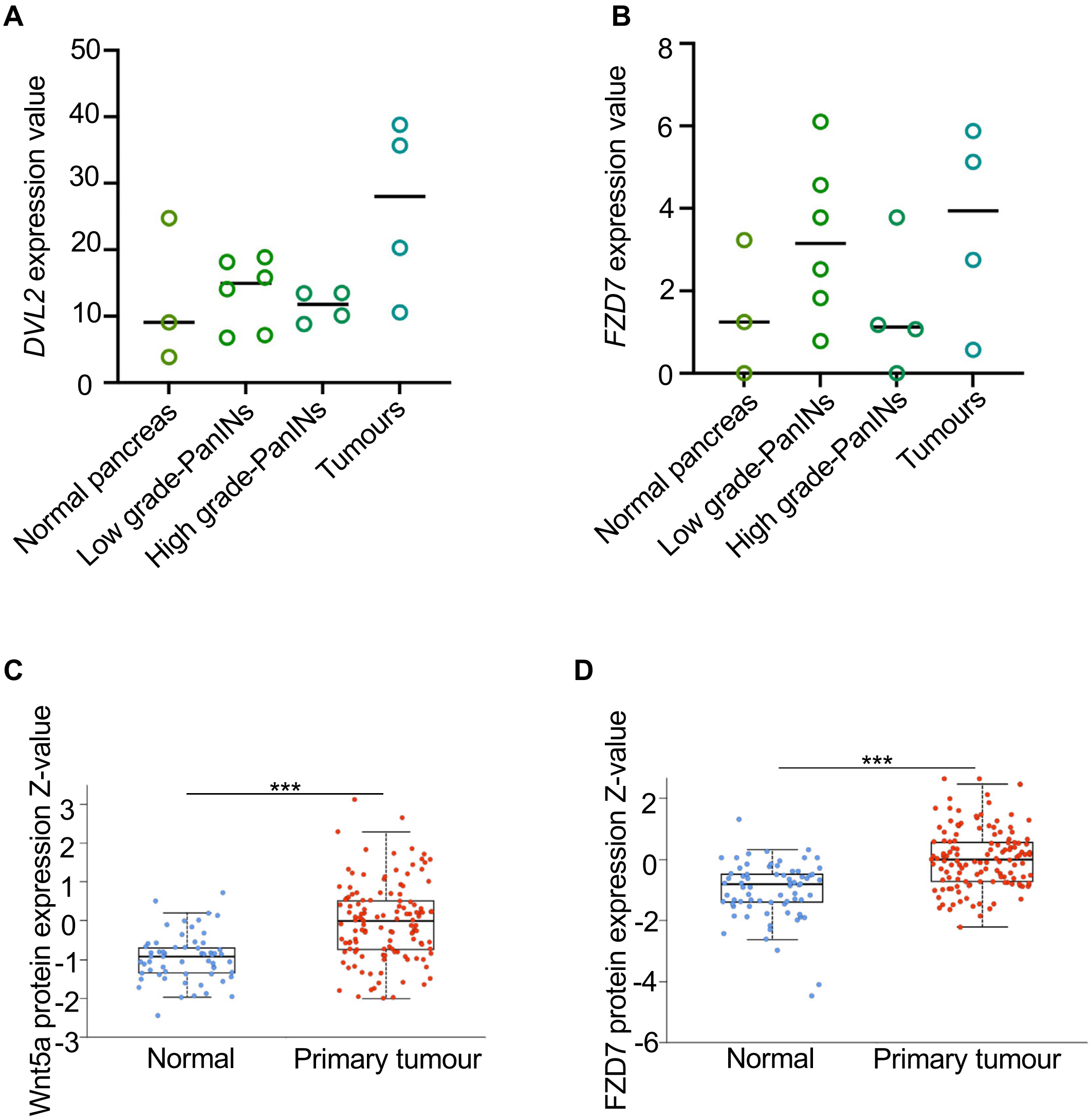

Supplementary Figure 10. Components of the WNT5A-ROR signaling pathway are up-regulated in human pancreatic cancer. *DVL2* (*A*) and *FZD7* (*B*) expression levels in normal pancreas, low-grade PanINs, high-grade PanINs, and tumors (GSE210351). (*C, D*) The *z* value of Wnt5a (*C*) or FZD7 (*D*) protein in normal pancreas (*blue*) and pancreatic adenocarcinoma (*red*). The *box plots* show individual values for each sample; normal n = 74; primary tumor n = 137. Student *t* tests were used to analyze the data. *$P = .032$; ***$P < .0001$. The data were obtained from the Clinical Proteomic Tumour Analysis Consortium.

Supplementary Table 14. List of Cell Stains and Antibodies Used

Antibody and fluorescence stains	Application	Antigen Retrieval	Dilution	Source	Identifier
Ki-67	IHC, IF	ProK 15’	1:500	Abcam	ab16667
CC-3	IHC	Citrate pH 6 15’	1:300	Cell Signaling	9661s
RFP	IHC, IF	ProK 15’	1:500	Rockland	600-401-379
Lectin PNA-A488	FACS	—		Invitrogen	L21409
c-Myc	WB	—	1:500	Santa Cruz	sc788
Phospho-p38	WB	—	1:1000	Cell Signaling	9211s
p38a	WB	—	1:1000	Santa Cruz	sc136210
Vinculin	WB	—	1:1000	Sigma Aldrich	V4505
Phospho-ERK	WB	—	1:1000	—	—
ERK	WB	—	1:1000	Cell Signaling	—
p21WAP/Cip	WB	—	1:1000	Sigma Aldrich	P1484
CD44	IF	ProK 15’	1:25	Cell Signaling	3570S
K-cadherin	IF	PoK 15’	1:50	ThermoFisher	MA1-06305
β-catenin	IF	Citrate pH 6 15’	1:50	BD Biosciences	610154
RFP	IF	Citrate pH 6 15’	1:500	Creative Diagnosis	DPATB-H83194
E-cadherin	IF	Citrate pH 6 15’	1:1000	BD Biosciences	610182
p27	IF	—	1:25	Santa Cruz	SC-528
Sox9	IF	—	1:100	ThermoFisher	PA5-81966
Wnt5a	IF	—	1:50	Santa Cruz	SC-30224
Dvl2	IF	—		Santa Cruz	SC-13974
goat anti-rabbit AF568	IF	—	1:1000	ThermoFisher	A-11011
donkey anti-mouse AF488		—	1:1000	ThermoFisher	A-21202
goat anti-rabbit AF488	IF	—	1:1000	ThermoFisher	A-11008
goat anti-rabbit HRP	WB	—	1:5000	Vector Laboratories	MP-7451-15
goat anti-mouse HRP	WB	—	1:5000	Vector Laboratories	MP-7802-15
Phalloidin	IF	—	1:1000	Sigma Aldrich	65906
E-cadherin	IF	—	1:100	ThermoFisher	13-1900
Caveolin	IF	—	1:50	Abcam	Ad2910
Hoechst	—	—	1:1000	ThermoFisher	H3570

FACS, fluorescence-activated cell sorted; IF, immunofluorescence; IHC, immunohistochemistry; WB, western blot.
